# Association of intrinsic capacity and neighborhood environment with dementia risk: an interaction and mediation analysis

**DOI:** 10.1093/geroni/igaf135

**Published:** 2025-12-13

**Authors:** Shuanglong Hou, Jing Luo, Rui Liu, Xueqiang Wang

**Affiliations:** Rehabilitation Medicine Center, The Second Affiliated Hospital of Wenzhou Medical University, Wenzhou, China; Department of Rehabilitation Medicine, Tangdu Hospital, Fourth Military Medical University, Xi’an, China; School of Rehabilitation Science, Shanghai University of Traditional Chinese Medicine, Shanghai, China; Department of Rehabilitation Medicine, Tangdu Hospital, Fourth Military Medical University, Xi’an, China; Rehabilitation Medicine Center, The Second Affiliated Hospital of Wenzhou Medical University, Wenzhou, China; Department of Rehabilitation Medicine, Wenzhou Medical University, Wenzhou, China

**Keywords:** CHARLS, Health equity, Mediation effect, Interaction effect

## Abstract

**Background and Objectives:**

While intrinsic capacity (IC) impairment and adverse neighborhood environments are established independent risk factors for dementia, their interaction effects and potential mediating pathways remain poorly understood. This study aimed to examine the independent, interactive, and mediating associations of IC, neighborhood environment, and dementia risk among middle-aged and older adults.

**Research Design and Methods:**

We analyzed data from 8,107 adults aged 50+ in the China Health and Retirement Longitudinal Study (2011–2020). IC was quantified using a composite impairment score encompassing locomotor, cognitive, sensory, psychological, and vitality domains. Neighborhood environment was classified by resource availability and social provisions (low risk; moderate risk; high risk). Cox proportional hazards models evaluated associations between IC, neighborhood environment, and dementia risk. The four-way decomposition model was used to examine the potential interaction and mediation effects of IC.

**Results:**

Over a median follow-up of 9 years, 909 incident dementia cases occurred. Adjusted analyses revealed dose-dependent relationships: each 1-point increase in IC impairment score elevated dementia risk by 26% (hazard ratio [HR] = 1.29, 95% confidence interval [CI]: 1.22–1.36). Compared with low-risk neighborhoods, moderate-risk (HR = 1.26, 95% CI: 1.08–1.47), and high-risk neighborhoods (HR = 1.41, 95% CI: 1.13–1.77) independently increased dementia risk. Four-way decomposition revealed the association between adverse neighborhood environments and increased dementia risk was partially explained by the pure mediation effect of IC, with no significant interaction-only/mediated interaction effects observed.

**Discussion and Implications:**

IC impairments and adverse neighborhood environments independently escalate dementia risk, with IC partially mediating the environmental effects. Integrating interventions targeting both individual capacity and community-level infrastructure may optimize dementia prevention strategies.

Innovation and Translational Significance: This study presents a novel integration of intrinsic capacity and neighborhood environment, using a nationwide cohort to quantify their independent, interaction, and mediation effects on dementia risk. Findings demonstrate that intrinsic capacity impairments and adverse neighborhood environments independently increase dementia risk, with no significant interaction effects observed. Furthermore, intrinsic capacity mediated the association between environmental exposures and dementia risk. The results highlight the importance of combining clinical intrinsic capacity screening with optimized community resource allocation. This evidence supports policy reforms targeting individual intrinsic capacity and environmental equity, offering feasible strategies to mitigate the global burden of dementia in aging populations.

## Background and objectives

Dementia represents an escalating global public health crisis. In 2019, approximately 57.4 million individuals lived with dementia, with projections reaching 152 million by 2050 (GBD 2019 Dementia Forecasting Collaborators, 2022). It entails significant cognitive decline, functional impairment, and memory loss, imposing substantial burdens on individuals, caregivers, and healthcare systems worldwide (Cheng, 2017; Tay et al., 2024). While advanced age and genetic susceptibility constitute established risks, an estimated 40%–45% of cases may be preventable through addressing modifiable risk factors (Livingston et al., 2020, 2024). The Lancet’s 2024 report identified 14 such modifiable risks operating across the lifespan (Livingston et al., 2024). Despite this, current prevention frameworks remain theoretically incomplete, inadequately accounting for the dynamic interplay between individuals’ physiological reserve and environmental context.

Contemporary understanding of healthy aging has shifted from pathology-centered models toward function-oriented frameworks. As defined by the World Health Organization (WHO), healthy aging is “the process of developing and maintaining the functional ability that enables wellbeing in older age,” fundamentally dependent on the ongoing interaction between an individual’s intrinsic capacity (IC) and their environment (Beard et al., 2016). IC represents the combination of all physical and mental capacities an individual can draw on at a given time, typically encompassing cognitive, locomotor, psychological, vitality, and sensory domains (Koivunen et al., 2022). Critically, emerging evidence identifies IC as a modifiable dementia risk factor, presenting novel opportunities for dementia prevention (Sun et al., 2024). Similarly, neighborhood environment has been demonstrated to exert significant influences on dementia risk: abundant community resources (Moored et al., 2025) and frequent social contact (Zhao et al., 2021) may reduce risk and enhance neurocognitive health, whereas neighborhood disadvantage (Becerril et al., 2023), social isolation (Choi et al., 2024), and environment barriers (Chen et al., 2022) exacerbate cognitive load and constitute independent risk factors.

Notwithstanding established independent effects of IC and neighborhood environment on dementia risk, substantial knowledge gaps persist. First, although conceptual frameworks (e.g., the WHO Healthy Aging framework [Beard et al., 2016] and the Selective Optimization with Compensation model [Donnellan & O’Neill, 2014]) emphasize individual-environment interactions, empirical evidence quantifying their synergistic, additive, or antagonistic joint effects on dementia risk remains limited. Characterizing such potential interactions is essential for a comprehensive understanding of environmental and individual risk landscapes. Second, while both IC and neighborhood environment operate on dementia risk, causal pathways connecting these factors are incompletely defined. A critical question is whether neighborhood environment impacts dementia risk, at least partially, through its influence on IC. Identifying IC’s potential mediating role is vital for elucidating underlying mechanisms and pinpointing intervention targets.

Therefore, leveraging data from a large-scale prospective cohort, this study aims to address these critical gaps. We hypothesize that (1) IC impairments and adverse neighborhood environments independently elevate dementia risk; (2) IC impairments and neighborhood environment exhibit significant interactions jointly influencing dementia risk; and (3) IC mediates the association between neighborhood environment and dementia risk. By integrating individual capacities and environmental contexts, this research provides a dual-perspective framework to inform multi-level preventive strategies targeting dementia risk reduction.

## Research design and methods

### Study design and population

This prospective cohort study utilized data from the China Health and Retirement Longitudinal Study (CHARLS), a nationally representative survey collecting health, economic status, family structure, and social support information among middle-aged and older Chinese adults (Zhao et al., 2014). Initiated in 2011, CHARLS conducted biennial to triennial follow-up interviews, with five data waves available up to now (2011, 2013, 2015, 2018, and 2020). In this study, exposures were assessed at baseline (2011), while incident dementia cases were tracked across four subsequent follow-up waves (2013–2020). We excluded participants with baseline dementia, missing key exposure information, or lost to follow-up. The final analytical cohort comprised 8,107 eligible participants, with a detailed selection process illustrated in Supplementary Figure 1 (see online supplementary material).

The CHARLS study protocol was approved by the Biomedical Ethics Review Committee of Peking University (IRB00001052-11015), and written informed consent was obtained from all participants. This study adheres to the Strengthening the Reporting of Observational Studies in Epidemiology (STROBE) guidelines.

### Assessment of intrinsic capacity

The concept of IC has been validated within the CHARLS cohort, demonstrating strong construct validity (Beard et al., 2022). Aligned with the WHO Integrated Care for Older People (ICOPE) framework (Beard et al., 2016) and established methodological protocols (Koivunen et al., 2022), IC was assessed across five dimensions: locomotion, cognition, sensory, psychological, and vitality capacity. Each domain was evaluated using standardized instruments, with impairments defined as follows: (1) The locomotion dimension was evaluated via the five-time chair stand test. A test duration > 12 s or inability to complete the test was defined as an impairment (Chen et al., 2020). (2) Cognition dimension was assessed using the Telephone Interview of Cognitive Status (TICS) scale, a 31-point instrument evaluating orientation, calculation, memory, and execution. Cognition impairment was defined as scores ≤ 1*SD* below the education-adjusted mean (Zhou et al., 2025). (3) Sensory dimension was assessed through self-reported vision and hearing status. Participants rated their near/distant eyesight and hearing ability, with sensory impairment defined as reporting “poor” in any category (Zhao et al., 2024). (4) Psychological dimension was evaluated using the 10-item Center for Epidemiological Studies Depression Scale (CES-D-10), with scores ≥10 indicating impairment (Luo et al., 2018). (5) Vitality dimension was assessed by maximum grip strength measured with a dynamometer. Sex-specific thresholds defined impairments as <28 kg for male and <18 kg for female (Chen et al., 2020).

According to the established methodology (Ramírez-Vélez et al., 2023; Sun et al., 2024), impairment in any domain contributed one point to the IC impairment index. The sensory domain comprises two distinct functional subdomains—vision and hearing—each considered necessary and independent for maintaining overall capacity (Sun et al., 2024; Zhou et al., 2025). Therefore, vision and hearing impairments were scored separately, yielding a theoretical index range of 0 to 6, with higher indices indicating greater impairment. Confirmatory factor analysis supported the theoretical structure, showing good model fit (CFI = 0.984, TLI = 0.920, RMSEA = 0.032, SRMR = 0.012). Due to the low frequency of cases with 4, 5, or 6 impairments, these categories were merged into a single group labeled “4+ impairments” to ensure statistical power. Thus, the IC impairment index was analyzed as an ordinal variable with five categories: 0, 1, 2, 3, and 4+ impairments.

### Assessment of neighborhood environment

The neighborhood environment was assessed by integrating both physical and social dimensions (Li, 2024; Moored et al., 2025; Tan et al., 2023; Zhang et al., 2025). The physical environment was measured through the accessibility of five key resources: safety resources (police stations), living resources (theaters, post offices, banks, farmer’s markets, supermarkets, and other recreational facilities), exercise resources (basketball courts, swimming pools, outdoor fitness equipment, table tennis facilities, chess/card rooms, or billiards rooms), medical resources (general/specialized/traditional Chinese medicine hospitals, pharmacies, community health centers/stations, township/village clinics), and service resources (nursing homes or senior activity centers). The social environment was operationalized via three indicators: community social organizations (e.g., calligraphy/painting associations, dance teams, senior associations, or organizations assisting vulnerable populations), minimum living allowances coverage, and pension availability for adults aged 65 years or above. Data were obtained from the CHARLS community questionnaires, completed by community or village leaders. Detailed operational definitions for each indicator are provided in Supplementary Table 2.

Each environmental characteristic was dichotomously coded (1 = unavailable, 0 = available). A composite index (range: 0–8) was constructed by summing all eight indicators. Neighborhood environments were then categorized into three levels: low risk (0–2 points), moderate risk (3–5 points), and high risk (6–8 points). This operational framework aligns with established epidemiological research methodologies (Yang et al., 2023; Zhang et al., 2024). To test robustness, sensitivity analyses were performed using environment stratifications derived from principal component analysis (PCA) alongside the primary equal-weight scoring approach. Additionally, given the potential for systematic disparities in resource accessibility between urban and rural settings, each indicator’s distribution was examined by residence type, confirming significant differences (Supplementary Table 3). Subsequent analyses incorporated stratification by urban–rural status.

### Outcome ascertainment

Dementia cases were identified by an algorithmic case definition based on coexistence of cognitive impairment and functional impairment, as well as self-reported (or caregiver-reported) physician-diagnosed dementia-related diseases (Liu et al., 2024; Zeng et al., 2025). This dual approach aligns with large-scale dementia prevalence surveys conducted in Chinese populations and is consistent with prior IMPACT-BAM studies (Ahmadi-Abhari et al., 2017; Chen et al., 2023). Cognitive function was assessed using the TICS; cognitive impairment was defined as a performance falling 1.5 *SD* or more below the age-specific mean (for individuals aged 50 and above) with the same educational stratum. Functional impairment was identified as requiring assistance or being unable to perform at least one basic activity of daily living, which included dressing, bathing, eating, getting into or out of bed, using the toilet, and controlling urination and defecation. Self-reported dementia cases were ascertained through an affirmative response to the question: “Have you been diagnosed with memory-related disease by a doctor?.” The follow-up duration was calculated from the baseline interview data until the earliest occurrence of either a dementia diagnosis (identified by either method above) or the last follow-up interview for censored cases.

### Potential covariates

Informed by recommendations from the Lancet commissions (Livingston et al., 2020) and prior epidemiological studies (Hendriks et al., 2024; Zheng et al., 2024), several sociodemographic characteristics and health-related covariates were considered. Sociodemographic factors included age, sex, residence (rural, urban), education level (0 year, 1–6 years, 7 years or higher), and marital status (married, others). Health-related lifestyle factors included smoking status (ever, never), drinking status (ever, never), daily sleep duration (<6, 6–8, or >8 hr/day), body mass index (BMI, <18.5, 18.5–23.99, or ≥24.0 kg/m^2^) and social isolation. Chronic diseases included body pain (none, 1–4, or ≥5 sites), hypertension, diabetes, heart disease, and stroke history. Social isolation was operationalized as no participation in social activities during the prior month. BMI was calculated from objectively measured height and weight (kg/m^2^). Trained interviewers collected other covariates using standardized questionnaires.

### Statistical analysis

Quantitative data with a normal distribution are presented as mean ± *SD*, and between-group comparisons were performed using one-way ANOVA. Categorical data are expressed as frequencies (percentages) and compared using chi-square tests. Missing data accounted for 3% (243/8107) of the total items; the patterns of missingness are detailed in Supplementary Table 1. Under the assumption that data were missing at random, multiple imputation by chained equations was employed to handle missing values. Hazard ratios (HRs) with 95% confidence intervals (CIs) from Cox proportional hazards models were calculated to estimate the associations of IC and neighborhood environment with dementia risk. Three hierarchical models were constructed: Model 1 was adjusted for age and sex; Model 2 was further adjusted for residence, marital status, education level, smoking status, drinking status, daily sleep time, BMI, and social isolation; and Model 3 was additionally adjusted for body pain, hypertension, diabetes, heart disease, and stroke history.

To evaluate the joint impact of IC and neighborhood environment on dementia risk and their potential interaction, the following analytical approach was employed. First, within each stratum of neighborhood environmental risk, the association between IC impairment and dementia risk was evaluated to examine whether the effect of IC varied by environmental risk levels. Second, participants were cross-classified into 15 mutually exclusive groups based on their levels of IC impairment and neighborhood environmental risk. Using the group with “no IC impairments residing in a low-risk neighborhood” as the reference, HRs and 95% CIs were calculated for the remaining 14 groups to illustrate the joint association of different risk combinations. Finally, to test for interaction effects, eight interaction terms were created by combining moderate- and high-risk neighborhoods with varying IC impairment levels. We assessed both multiplicative interactions and additive interactions by calculating the relative excess risk due to interaction (RERI), the attributable proportion due to interaction (AP), and the synergy index (SI) (Knol et al., 2011).

**Figure 1.**
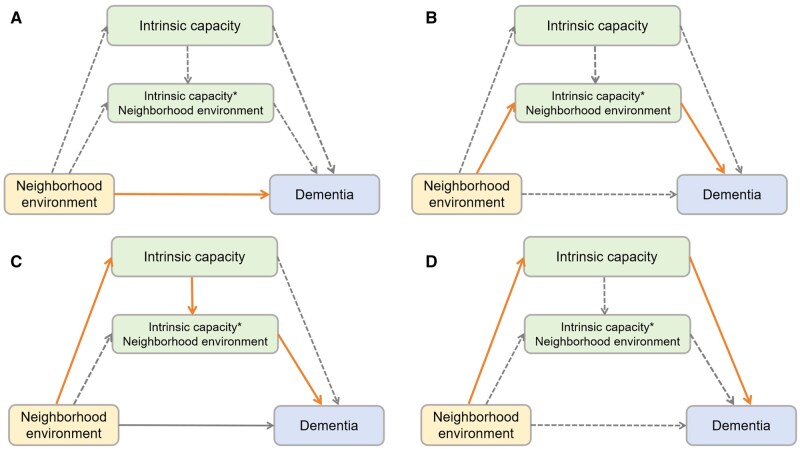
Four-way decomposition diagram for the association of neighborhood environment and intrinsic capacity with dementia risk. (A) controlled direct effect; (B) reference interaction; (C) mediated interaction; (D) pure indirect effect. The sum of these four components equals the total effect.

To gain deeper insight into the complex relationships among variables, we performed a four-way decomposition of the total effect. Supplementary Figure 1 illustrates the hypothesized causal pathways of this study using a directed acyclic graph. As illustrated in Figure 1, this approach decomposes the total effect into four distinct components (Discacciati et al., 2018): (1) Controlled direct effect (CDE): The direct effect of the neighborhood environment on dementia risk, independent of mediation or interaction with IC. (2) Reference interaction effect (INTref): The effect due to interaction between the neighborhood environment and IC, without mediation. (3) Mediated interaction effect (INTmed): The effect resulting from the interaction between the neighborhood environment and IC, coupled with the mediation effect. (4) Pure indirect effect (PIE): The effect of the neighborhood environment on dementia risk that is solely mediated by IC, in the absence of any interaction. After full adjustment for covariates, the total effect, each component-specific effect, and their 95% CIs were estimated. Furthermore, the incremental predictive value of IC, neighborhood environment, and their combination was evaluated by calculating Harrell’s C-index, continuous net reclassification improvement (NRI), categorical NRI, and the integrated discrimination improvement (IDI). For the categorical NRI, a three-tier risk classification was used with thresholds set at <5% (low risk), 5–10% (medium risk), and >10% (high risk) (Leening et al., 2014).

**Figure 2.**
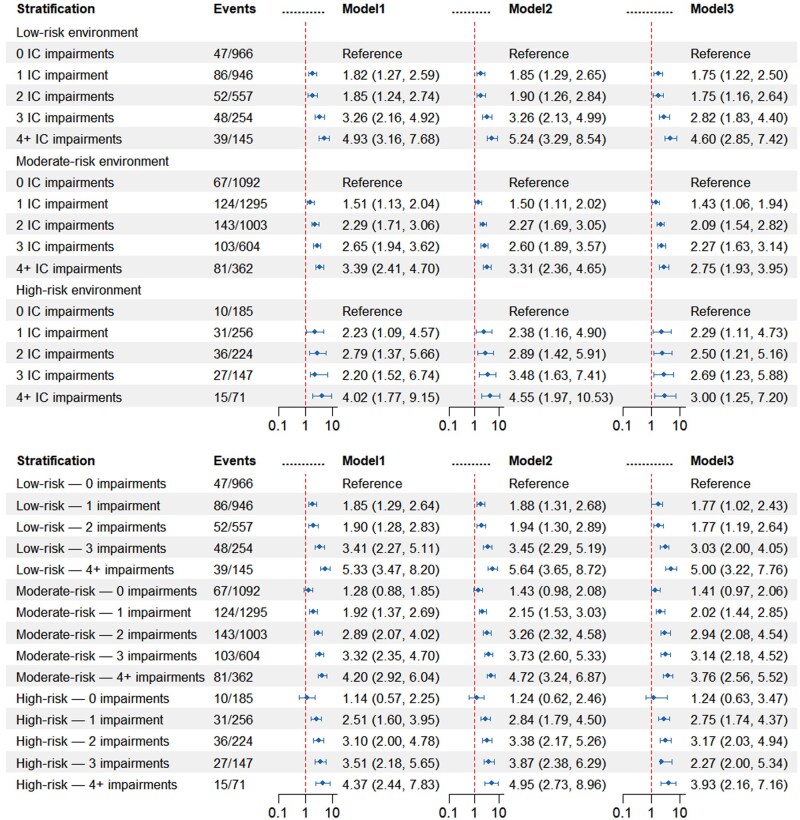
Joint associations of intrinsic capacity and neighborhood environment with dementia risk. IC = intrinsic capacity. Model 1: Adjusted for age and sex. Model 2: Adjusted for age, sex, residence, marital status, education level, drinking status, smoking status, body mass index, and sleep time. Model 3: Additionally adjusted for body pain, hypertension, diabetes, heart disease, and stroke.

To identify potential effect modifiers, subgroup analyses were conducted by age (50–59 years vs ≥60 years), sex (male vs female), and residence (urban vs rural). A series of sensitivity analyses were performed to examine the robustness of the findings: (1) using a complete-case dataset without multiple imputation to assess the impact of missing data; (2) excluding incident dementia cases identified during the second and third follow-up waves to minimize potential reverse causality; (3) distinguishing between algorithm-defined and self-reported dementia cases to evaluate misclassification bias; (4) calculating E-values to quantify the strength of association required for an unmeasured confounder would need to explain away the observed associations; (5) merging vision and hearing scores into a single sensory domain indicator (range: 0–5) to test the influence of overweighting the sensory dimension; and (6) replacing the equal-weight environmental index with a PCA-based weighted index to assess the impact of alternative construct methods.

All statistical analyses were performed using SPSS 25.0, Stata 17.0, and R 4.4.0. A two-tailed *p* value <.05 was considered statistically significant.

## Results

### Baseline characteristics of study population

Of the 17,705 participants enrolled at baseline, 9,598 were excluded for non-compliance with inclusion criteria. Baseline characteristics for included and excluded participants are detailed in Supplementary Table 4. Table 1 presents baseline characteristics stratified by IC impairments. The 8,107 eligible participants had a mean age of 61.4 ± 7.6 years, with 50% being male. Compared with participants without IC impairments, those with severe IC impairments were significantly older and exhibited significantly higher proportions of women, rural residents, individuals with low educational levels, and those experiencing social isolation, body pain, hypertension, diabetes, heart disease, and stroke history. Baseline characteristics stratified by neighborhood environment categories are presented in Supplementary Table 5.

**Table 1.** Baseline characteristics of participants, stratified by intrinsic capacity impairment.

Characteristics	**Overall** **(*N = *8,107)**	IC impairments					*p* value
		**0 (*n = *2,243)**	**1 (*n = *2,497)**	**2 (*n = *1,784)**	**3 (*n = *1,005)**	**4+ (*n = *578)**	
**Age, years**	61.4 ± 7.6	59.8 ± 6.7	60.8 ± 7.3	61.7 ± 7.7	63.3 ± 8.1	66.0 ± 9.1	<.001
**Sex**							<.001
** Male**	4,050 (50.0)	1,327 (59.2)	1,307 (52.3)	801 (44.9)	395 (39.3)	220 (38.1)	
** Female**	4,057 (50.0)	916 (40.8)	1,190 (47.7)	983 (55.1)	610 (60.7)	358 (61.9)	
**Residence**							
** Rural**	6,441 (79.4)	1,625 (72.4)	1,940 (77.7)	1,485 (83.2)	884 (88.0)	507 (87.7)	
** Urban**	1,666 (20.6)	618 (27.6)	557 (22.3)	299 (16.8)	121 (12.0)	71 (12.3)	
**Marital status**							<.001
** Married**	6,738 (83.1)	1,976 (88.1)	2,107 (84.4)	1,457 (81.7)	776 (77.2)	422 (73.0)	
** Others**	1,369 (16.9)	267 (11.9)	390 (15.4)	327 (18.3)	229 (22.8)	156 (27.0)	
**Education level**							<.001
** Low**	2,349 (29.0)	428 (19.1)	674 (27.0)	571 (32.0)	393 (39.1)	283 (49.0)	
** Moderate**	3,472 (42.8)	909 (40.5)	1,104 (44.2)	800 (44.8)	438 (43.6)	221 (38.2)	
** High**	2,286 (28.2)	906 (40.4)	719 (28.8)	413 (23.2)	174 (17.3)	74 (12.8)	
**Smoking status**							<.001
** Ever**	3,413 (42.1)	1,050 (46.8)	1,070 (43.1)	709 (39.7)	368 (36.6)	209 (36.2)	
** Never**	4,694 (57.9)	1,193 (53.2)	1,420 (56.9)	1,075 (60.3)	637 (63.4)	369 (63.8)	
**Drinking status**							<.001
** Ever**	2,740 (33.8)	910 (40.6)	873 (35.0)	555 (31.1)	266 (26.5)	136 (23.5)	
** Never**	5,367 (66.2)	1,333 (59.4)	1,624 (65.0)	1,229 (68.9)	739 (73.5)	442 (76.5)	
**Sleeping time**							<.001
** <6 hr**	2,443 (30.1)	437 (19.5)	658 (26.4)	648 (36.3)	418 (41.6)	282 (48.8)	
** 6–8 hr**	5,020 (61.9)	1,625 (72.4)	1,656 (66.3)	997 (55.9)	488 (48.6)	284 (43.9)	
** >8 hr**	644 (7.9)	181 (8.1)	183 (7.3)	139 (7.8)	99 (9.8)	42 (7.3)	
**BMI, kg/m^2^**							<.001
** <18.5**	535 (6.6)	76 (3.4)	170 (6.8)	133 (7.5)	90 (9.0)	66 (11.4)	
** 18.5–23.9**	4,407 (54.4)	1,212 (54.0)	1,329 (53.2)	990 (55.5)	555 (55.2)	321 (55.5)	
** ≥24.0**	3,165 (39.0)	955 (42.6)	998 (40.0)	661 (37.1)	360 (35.8)	191 (33.0)	
**Social isolation**	3,988 (47.2)	935 (41.7)	1,218 (48.8)	903 (50.6)	588 (50.5)	344 (59.5)	<.001
**Pain**							<.001
** 0 site**	5,412 (66.8)	1,932 (86.1)	1,798 (72.0)	998 (55.9)	452 (45.0)	232 (40.1)	
** 1–4 sites**	1,716 (21.2)	243 (10.8)	484 (19.4)	503 (28.2)	311 (30.9)	175 (30.3)	
** ≥5 sites**	979 (12.1)	68 (3.0)	215 (8.6)	283 (15.9)	242 (24.1)	171 (29.6)	
**Hypertension**	2,096 (25.9)	506 (22.6)	618 (24.7)	479 (26.8)	304 (30.2)	189 (32.7)	<.001
**Diabetes**	449 (5.1)	96 (4.3)	165 (6.6)	95 (5.3)	76 (7.6)	56 (9.7)	<.001
**Heart disease**	894 (10.2)	180 (8.0)	275 (11.0)	244 (13.7)	181 (18.0)	120 (20.8)	<.001
**Stroke**	145 (1.8)	16 (0.7)	38 (1.5)	41 (2.3)	29 (2.7)	21 (3.6)	<.001

*Note*. BMI = body mass index; IC = intrinsic capacity.

### Independent association of intrinsic capacity and neighborhood environment with dementia risk

During a median follow-up of 9.0 years, 909 incident cases were identified, corresponding to an incidence rate of 11.2%. Table 2 presents the associations between IC impairments, neighborhood environment, and dementia risk. In the fully-adjusted model, participants with 1, 2, 3, and 4+ impairments showed significantly elevated HRs for dementia compared with those without impairments: 1.64 (95% CI: 1.31–2.04), 2.11 (95% CI: 1.68–2.64), 2.54 (95% CI:1.98–3.25), and 3.29 (95% CI: 2.52–4.31), respectively. When analyzed as a continuous variable, each 1-point increase in the IC impairment index was associated with a 29% elevated risk of dementia (HR = 1.29; 95% CI: 1.23–1.36). Similarly, neighborhood environment risk was significantly associated with dementia risk. Compared with participants residing in low-risk neighborhoods, those in moderate-risk (HR = 1.26, 95% CI: 1.08–1.47) and high-risk (HR = 1.41, 95% CI: 1.13–1.77) environments exhibited significantly higher dementia risks. Each 1-point increase in the composite neighborhood environment index was associated with an 11% increase in dementia risk (HR = 1.08, 95% CI: 1.04–1.12).

**Table 2.** Independent associations of intrinsic capacity and neighborhood environment with dementia risk.

Variables	Event No.	HR (95% CI)		
		**Model 1^a^**	**Model 2^b^**	**Model 3^c^**
**IC impairment index**				
** 0**	124/2,243	Reference	Reference	Reference
** 1**	241/2,497	1.71 (1.37, 2.12)***	1.72 (1.38, 2.14)***	1.64 (1.31, 2.04)***
** 2**	231/1,784	2.28 (1.83, 2.84)***	2.31 (1.85, 2.89)***	2.11 (1.68, 2.64)***
** 3**	178/1,005	2.95 (2.34, 3.73)***	2.97 (2.34, 3.77)***	2.54 (1.98, 3.25)***
** 4+**	135/578	3.93 (3.06, 5.06)***	4.02 (3.10, 5.21)***	3.29 (2.52, 4.31)***
** Per 1-point increase**	909/8,107	1.34 (1.28, 1.41)***	1.35 (1.28, 1.42)***	1.29 (1.22, 1.36)***
**Neighborhood environment**				′
** Low-risk**	272/2,868	Reference	Reference	Reference
** Moderate-risk**	518/4,356	1.26 (1.09, 1.46)***	1.32 (1.13, 1.54)***	1.26 (1.08, 1.47)**
** High-risk**	119/883	1.42 (1.15, 1.76)***	1.46 (1.17, 1.82)**	1.41 (1.13, 1.77)**
** Per 1-point increase**	909/8,107	1.08 (1.04, 1.12)***	1.09 (1.05, 1.13)***	1.08 (1.04, 1.12)***

*Note*. IC = intrinsic capacity.

a Model 1: Adjusted for age and sex.

b Model 2: Adjusted for age, sex, residence, marital status, education level, drinking status, smoking status, BMI, and sleep time.

c Model 3: Additionally adjusted for body pain, hypertension, diabetes, heart disease, and stroke based on Model 2.

** *p *< .01.

*** *p *< .001.

### Joint and interactive association of intrinsic capacity and neighborhood environment with dementia risk

Figure 2 presents the joint effects of IC impairments and neighborhood environment on dementia risk. A consistent association between IC impairments and dementia risk was observed across all strata of neighborhood environmental risk (all *p *< .05). Participants were classified into 15 groups according to their status of IC impairment and neighborhood environmental risk. In comparison to those without IC impairments living in low-risk neighborhoods, most combined groups exhibited an elevated dementia risk. Notably, the highest risk was observed among participants with 4 or more IC impairments residing in low-risk neighborhood environments (HR = 5.00; 95% CI: 3.22–7.76). As shown in Supplementary Table 6, eight different interaction terms were evaluated. No significant multiplicative or additive interaction was detected for any term, indicating that the effects of IC and neighborhood environment on dementia risk are largely independent.

### Four-way decomposition of the effects of neighborhood environment and intrinsic capacity on dementia risk

Table 3 presents the results of the four-way decomposition analysis examining the effects of neighborhood environment and IC on dementia risk. Compared to low-risk neighborhood environments, moderate-risk environments demonstrated a TE of 0.255 (95% CI: 0.058–0.451, *p *= .011) on dementia risk. The CDE was 0.234 (95% CI: 0.038–0.429, *p *= .019), and the PIE was 0.061 (95% CI: 0.035–0.087, *p *< .001). Neither the INTref nor the INTmed reached statistical significance (both *p *>.05). In the comparison between high-risk and low-risk neighborhood environments, the TE was 0.522 (95% CI: 0.165–0.879, *p *= .004), the CDE was 0.477 (95% CI: 0.110–0.845, *p *= .011), and the PIE was 0.105 (95% CI: 0.060–0.150, *p *< .001). Similarly, neither INTref nor INTmed showed statistically significant effects (both *p *> .05). These results indicate that the association between neighborhood environmental risk and dementia risk is primarily attributable to both direct (CDE) and indirect (PIE) effects, with no significant interactive components observed.

**Table 3.** Four-way decomposition of the effects of neighborhood environment and intrinsic capacity with dementia risk.

Comparison group	Effect component	Est (95% CI)	*SE*	*p* value
**Moderate-risk vs low-risk**	TE	0.255 (0.058, 0.451)	0.100	.011
	CDE	0.234 (0.038, 0.429)	0.100	.019
	INTref	−0.027 (−0.058, 0.004)	0.016	.089
	INTmed	−0.013 (−0.035, 0.009)	0.011	.241
	PIE	0.061 (0.035, 0.087)	0.014	<.001
**High-risk vs low-risk**	TE	0.522 (0.165, 0.879)	0.182	.004
	CDE	0.477 (0.110, 0.845)	0.188	.011
	INTref	−0.034 (−0.074, 0.006)	0.021	.097
	INTmed	−0.026 (−0.091, 0.039)	0.0330	.429
	PIE	0.105 (0.060, 0.150)	0.0230	<.001

*Note*. CDE = controlled direct effect; INTref = reference interaction; INTmed = mediated interaction; PIE = pure indirect effect; TE = total effect. All pathways were adjusted for age, sex, residence, marital status, education level, drinking status, smoking status, body mass index, sleep time, body pain, hypertension, diabetes, heart disease, and stroke.

### Incremental predictive value of intrinsic capacity and neighborhood environment on dementia risk

Supplementary Table 7 shows the incremental predictive value of IC and neighborhood environment for dementia risk. Based on C-statistic, IDI, and NRI results, incorporating either IC, neighborhood environment, or both into the base model significantly improved predictive performance. The greatest improvements were observed when both IC and neighborhood environment were incorporated into the base model, yielding an IDI of 0.019 (95% CI: 0.013–0.029, *p *< .001), a continuous NRI of 0.134 (95% CI: 0.088–0.180, *p *< .001), and an increase in the C-statistic from 0.680 to 0.714 (*p *< .001). These results indicate that both IC and neighborhood provide independent incremental predictive value for dementia risk. Their combined inclusion significantly enhances risk discrimination and reclassification accuracy.

### Subgroup and sensitivity analysis

As shown in Supplementary Table 8, the association between IC impairments and dementia risk remained consistent across all subgroups, including age (50–59 years and ≥60 years), sex (male and female), and residence (rural and urban). However, the association between neighborhood environment and dementia risk was not significant among urban residents, though it remained consistent across all other subgroups. Furthermore, both joint analyses (Supplementary Table 9) and four-way decomposition analyses (Supplementary Table 10) revealed similar association patterns across these subgroups.

Sensitivity analyses were conducted to assess the robustness of the findings. Using unimputed complete-case data (Supplementary Tables 11–13), excluding incident dementia cases identified during the second and third follow-up waves (Supplementary Tables 14–16), or distinguishing between self-reported and algorithm-defined dementia cases (Supplementary Tables 17–19), we repeated analyses of the independent associations, joint associations, and four-way decomposition models. The results showed that all association patterns remained highly consistent with the primary findings, indicating robustness to missing data, potential reverse causality, and variations in dementia case definitions. The calculated E-values for the independent associations were substantial, suggesting that any unmeasured confounder would need strong associations with both exposure and outcome to explain away the observed effects (Supplementary Table 20). Moreover, merging vision and hearing scores within the sensory domain did not alter the strength or direction of the association between IC impairments and dementia risk, confirming that the main findings are insensitive to this scoring modification (Supplementary Table 21). Finally, when a weighted environmental index was used to reclassify neighborhood risk levels, the association with dementia risk remained highly consistent with that from the original equal-weight index, supporting the rationality of applying an equal-weight approach for the composite neighborhood environment index (Supplementary Table 22).

## Discussion and implications

This nationwide prospective cohort study investigated the independent, interactive, and mediating associations of IC and neighborhood environment with dementia risk among middle-aged and older adults. Our findings robustly demonstrate that both IC impairment and adverse neighborhood environments independently predicted an elevated dementia risk, with clear dose–response gradients observed. Notably, although no significant interaction effects were detected, IC served as a significant mediator in the association between neighborhood environment and dementia risk. Furthermore, incorporating both IC and neighborhood environment measures significantly enhanced the predictive performance of the base model. While the absolute improvement in the C-index was modest, the significant categorical NRI underscores its clinical utility for risk stratification at the population level.

Our study corroborates and expands prior evidence identifying IC as a pivotal risk factor for dementia. A clear dose–response relationship was observed, with each additional impairment associated with a 26% increased risk, and severe impairment (≥4 impairments) corresponding to a threefold risk elevation. This aligns with cohort studies suggesting that functional decline predicts elevated dementia risk (Sun et al., 2024) and is consistent with research linking specific domains (e.g., cognitive function, grip strength, or depressive symptoms) to dementia in diverse populations (Collyer et al., 2022; Kuo et al., 2023; Rubin, 2018). Importantly, by employing an integrated IC construct rather than focusing on single domains, our study provides a more comprehensive evaluation of physiological reserve and demonstrates stronger predictive strength. Mechanistically, IC impairments may reflect underlying neuropathology (e.g., neuroinflammation, oxidative stress, or mitochondrial dysfunction) or diminish cognitive reserve, rendering the brain less able to compensate for dementia-related pathological alterations (López-Ortiz et al., 2024; Lu et al., 2023). These findings support the implementation of the WHO’s ICOPE framework, highlighting its potential for dementia risk stratification in clinical and public health settings. The integrated IC approach may enable earlier identification of at-risk individuals compared to domain-specific assessments alone.

The independent association between neighborhood environment and dementia risk reinforces the importance of contextual factors in cognitive aging. We operationalized neighborhood environment through a composite index encompassing both built (e.g., safety resources, medical facilities) and social (e.g., social organizations, pension provisions) dimensions, and observed significant urban-rural disparities in resource availability. Participants in high-risk neighborhood environments faced a 41% increased dementia risk compared to those in low-risk environments. These findings corroborate prior research linking neighborhood-level factors to dementia risk, including neighborhood disadvantage (Mobley et al., 2023), low social cohesion (Fujihara et al., 2023), and limited access to resources such as sports facilities (Moored et al., 2025), and sidewalks (Tani et al., 2021). Limited resource availability may restrict opportunities for cognitive stimulation, physical activity, social engagement, and timely healthcare access, all of which are implicated in the pathogenesis of dementia. Subgroup analyses stratified by urban–rural residence confirmed the robustness of the observed associations and further demonstrated their pronounced strength in resource-limited rural settings. These findings underscore the need to integrate neighborhood environmental factors into dementia prevention strategies, particularly in light of rapid urbanization and disparities in community resource allocation across China. Such integration supports the development of targeted policies and interventions designed to explicitly address equity gaps in population health.

No statistically significant multiplicative or additive interactions were detected, and the possibility that this may be due to a limited sample size or number of events, resulting in insufficient statistical power, cannot be entirely ruled out. However, the four-way decomposition results also indicated no significant interaction effects, suggesting that the impacts of IC impairment and adverse neighborhood environment on dementia risk operate largely independently. Notably, integrating both IC and neighborhood environment indicators into the base model significantly improved its predictive performance. This indicates that a combined assessment can improve the accurate classification of dementia risk at the population level, aiding the optimization of public health resource allocation. Furthermore, the significant PIE observed suggests a reasonable mediating pathway: the neighborhood environment may partially influence dementia risk through its effect on IC. Within an environmental context, residing in a resource-poor neighborhood may gradually deplete an individual’s functional reserves by restricting opportunities for physical activity, social participation, and accessibility to health-promoting services, thereby increasing susceptibility to dementia (Sun et al., 2024; Yu et al., 2024; Zhao et al., 2024). Mechanistically, adverse environmental exposures may contribute to physiological dysregulation, such as systemic inflammation (Giurgescu et al., 2019), neuroendocrine stress responses(Evans et al., 2020), or metabolic disturbances (Deng et al., 2024), which can lead to impairments across IC domains. Ultimately, these pathways collectively elevate the risk of dementia onset.

Our findings hold significant implications for developing dementia prevention strategies. First, the graded risk associated with IC impairment highlights the urgency of integrating IC screening within routine primary care for middle-aged and older adults. Early identification of declining domains enables targeted interventions, including multi-modal strategies combining exercise rehabilitation and nutritional support (Ferrara et al., 2023; Valenzuela et al., 2025). Second, identified environmental risk factors necessitate multi-level, environmentally supportive community interventions. Urban planning and social policies should prioritize equitable access to safe environments, healthcare, recreational facilities, social security, and programs promoting social inclusion. Third, the observed mediating effects and joint risks suggest that integrating individual-level capacity-building interventions with community-level environmental enhancements could yield synergistic benefits, particularly for vulnerable individuals residing in resource-poor areas. Additionally, targeted community environment optimization can serve as an effective strategy for enhancing specific IC-related domains. For instance, improving cognition by fostering social interaction (Nakagomi et al., 2023), enhancing psychological capacity by reducing social isolation and creating opportunities for connection (Nakagomi et al., 2023), and boosting locomotion by increasing physical activity facilities (Moored et al., 2025) may help mitigate the challenges associated with large-scale community environment renovations in the short term.

Study strengths include its large, nationally representative cohort of middle-aged and older adults, prospective design, extended follow-up, rigorous adjustment for key confounders, and comprehensive sensitivity analyses. However, several limitations warrant consideration. First, residual confounding by unmeasured factors (e.g., dietary patterns and APOE ε4 status; Sun et al., 2024) cannot be fully excluded, although E-values suggest relative robustness of the observed associations. Second, neighborhood environment characteristics relied on self-reported data; future studies could benefit from objective community audits. Dementia cases were identified using a combination of algorithmic definitions and self-report, which, although widely applied (Liu et al., 2024; Zeng et al., 2025), clinically confirmed diagnoses are preferable for subsequent studies. Third, IC was measured only at baseline, which limits the strength of causal inference in the mediation pathway. Future investigations should incorporate longitudinal IC data and causal inference methods to address this limitation. Additionally, the neighborhood environment index was constructed using equal weights for all components, which may not reflect potential differences in the contributions of individual resources. However, a sensitivity analysis employing a weighted index derived from PCA produced results consistent with the primary findings. Finally, findings specific to the Chinese context may not be fully generalizable to other societal contexts, particularly regarding neighborhood resource definitions and availability.

## Conclusions

This study robustly demonstrates that impairments in IC and exposure to adverse neighborhood environments independently elevate dementia risk, with IC partially mediating the environmental influence. These findings highlight the importance of adopting integrated, multi-level preventive strategies that simultaneously target the enhancement of individual functional reserves and neighborhood environments. Implementing such strategies holds promise for promoting healthy brain aging and mitigating the global burden of dementia through concerted public health and policy actions.

## Supplementary Material

igaf135_Supplementary_Data

## Data Availability

The data, analytic methods, or materials of this paper are available to other researchers for replication purposes. The datasets are available for download at the CHARLS home website: http://charls.pku.edu.cn. The author acknowledges that this is not a preregistered study.
