# Supplementary material for: Association of intrinsic capacity and neighborhood environment with dementia risk: an interaction and mediation analysis
Source: Innov Aging. 2025 Dec 13;10(2):igaf135. doi: 10.1093/geroni/igaf135 (PMC12907018; doi:10.1093/geroni/igaf135)
Supplement: igaf135_Supplementary_Data [file igaf135_supplementary_data.docx]

***Innovation in Aging* Supplementary Material:** **Hou, Luo, Liu, & Wang. Association of intrinsic capacity and neighborhood environment with dementia risk: an interaction and mediation analysis.**

**Supplementary Table 1.** Amount and proportion of missing data for each covariate

| **Characteristics** | **Amount and proportion** | **Characteristics** | **Amount and proportion** |
| --- | --- | --- | --- |
| Age | 0 (0.00%) | Sex | 0 (0.00%) |
| Residence | 2 (0.02%) | Marital status | 0 (0.00%) |
| Education level | 0 (0.00%) | Smoking status | 1 (0.01%) |
| Drinking status | 0 (0.00%) | Sleeping time | 51 (0.00%) |
| BMI | 72 (0.89%) | Social isolation | 0 (0.00%) |
| Body pain | 0 (0.00%) | Hypertension | 31 (0.38%) |
| Diabetes | 68 (0.83%) | Heart disease | 40 (0.49%) |
| Stroke | 18 (0.22%) |  |  |

*Note*. BMI = Body mass index.

**​**

**Supplementary Table 2.** Operational definition of neighborhood environment

| **Subfactors** | **Question** | **Code** |
| --- | --- | --- |
| Safety resource | How many police stations are there in your village/ community? | None = 1; one or more = 0 |
| Service resource | How many nursing homes or activity centers for the elderly are there in your village /community? | None = 1; one or more = 0 |
| Living resource | How many theaters, post offices, banks, farmer’s markets, supermarkets, or other recreational facilities are there in your village /community? | None = 1; one or more = 0 |
| Exercise resource | Does your village /community have basketball courts, swimming pools, outdoor fitness equipment, table tennis facilities, chess/card rooms, or billiards rooms? | None = 1; yes, one or more = 0 |
| Medical resource | Does your village/community have traditional Chinese medicine hospitals, pharmacies, community health centers/stations, or township/village clinics? | None = 1; yes, one or more = 0 |
| Social organization | Does your village/community have calligraphy/painting associations, dance teams, senior associations, or organizations assisting vulnerable populations? | None = 1; yes, one or more = 0 |
| Living allowance | Does your village/community have minimum living allowance? | None = 1; yes = 0 |
| Pension | Does your village/community issue pension to persons older than 65? | None = 1; yes = 0 |

**Supplementary Table 3.** A comparison of neighborhood environments in urban and rural settings

|  | **Rural** | **Urban** | ***p* value** |
| --- | --- | --- | --- |
| Neighborhood environment scores | 3.6±1.7 | 2.3±1.7 | < 0.001 |
| Neighborhood environment categorization |  |  | < 0.001 |
| Low-risk | 1807 (28.1) | 1061 (63.7) |  |
| Moderate-risk | 3839 (59.6) | 571 (31.0) |  |
| High risk | 795 (12.3) | 88 (5.3) |  |
| Safety resource |  |  | < 0.001 |
| Available | 2112 (32.8) | 1214 (72.9) |  |
| Unavailable | 4329 (67.2) | 452 (27.1) |  |
| Service resource |  |  | < 0.001 |
| Available | 2051 (31.8) | 1095 (65.7) |  |
| Unavailable | 4390 (68.2) | 571 (34.3) |  |
| Living resource |  |  | < 0.001 |
| Available | 6073 (94.3) | 1622 (97.4) |  |
| Unavailable | 368 (5.7) | 44 (2.6) |  |
| Exercise resource |  |  |  |
| Available | 3462 (53.7) | 1303 (78.2) |  |
| Unavailable | 2979 (46.3) | 363 (21.8) |  |
| Medical resource |  |  | < 0.001 |
| Available | 5210 (80.9) | 1223 (73.4) |  |
| Unavailable | 1231 (19.1) | 443 (26.6) |  |
| Social organization |  |  | < 0.001 |
| Available | 2750 (42.7) | 1285 (77.1) |  |
| Unavailable | 3691 (57.3) | 381 (22.9) |  |
| Living allowance |  |  | < 0.001 |
| Available | 5140 (79.8) | 1475 (88.5) |  |
| Unavailable | 1301 (20.2) | 151 (11.5) |  |
| Pension |  |  | 0.044 |
| Available | 1491 (21.3) | 347 (20.8) |  |
| Unavailable | 4950 (78.7) | 1319 (79.2) |  |

**Supplementary Table 4.** Baseline characteristics between included and excluded participants

| **Characteristics** | **Total (*N*=17705)** | **Included (*n*=8107)** | **Excluded (*n*=9598)** | ***p* value** |
| --- | --- | --- | --- | --- |
| Age, years | 59.1±10.2 | 61.4±7.6 | 57.1±11.5 | < 0.001 |
| Missing | 54 (0.3) | 0 (0.0) | 54 (0.1) |  |
| Sex |  |  |  | < 0.001 |
| Male | 8477 (47.9) | 4050 (50.0) | 4427 (46.1) |  |
| Female | 9221 (52.1) | 4057 (50.0) | 5164 (53.8) |  |
| Missing | 7 (0.0) | 0 (50.0) | 7 (0.1) |  |
| Residence |  |  |  |  |
| Rural | 13694 (77.4) | 6441 (79.5) | 7253 (75.6) |  |
| Urban | 3979 (22.5) | 1664 (20.5) | 2315 (24.1) |  |
| Missing | 32 (0.2) | 2 (0.0) | 30 (0.3) |  |
| Marital status |  |  |  | < 0.001 |
| Married | 14170 (80.1) | 6738 (83.1) | 7432 (77.4) |  |
| Others | 3505 (19.8) | 1369 (16.9) | 2136 (22.3) |  |
| Missing | 30 (0.1) | 0 (0.0%) | 30 (0.3) |  |
| Education level |  |  |  | < 0.001 |
| Low education level | 4803 (27.1) | 2349 (29.0) | 2454 (25.6) |  |
| Moderate education level | 6952 (39.3) | 3472 (42.8) | 3480 (36.3) |  |
| High education level | 5898 (33.3) | 2286 (28.2) | 3612 (37.6) |  |
| Missing | 52 (0.3) | 0 (0.0) | 52 (0.5) |  |
| Smoking status |  |  |  | < 0.001 |
| Never | 10626 (60.0) | 3413 (42.1) | 5933 (61.8) |  |
| Ever | 6931 (39.1) | 4693 (57.9) | 3518 (36.7) |  |
| Missing | 148 (0.8) | 1 (0.0) | 147 (1.5) |  |
| Drinking status |  |  |  | < 0.001 |
| Never | 11783 (66.6) | 5367 (66.2) | 6416 (66.9) |  |
| Ever | 5767 (32.6) | 2740 (33.8) | 3027 (31.5) |  |
| Missing | 155 (0.9) | 0 (0.0) | 155 (1.6) |  |
| Sleeping status |  |  |  | 0.001 |
| < 6 hours | 4688 (26.5) | 2430 (30.0) | 2258 (23.5) |  |
| 6-8 hours | 10010 (56.5) | 4992 (61.6) | 5018 (52.3) |  |
| ＞8 hours | 1373 (7.8) | 634 (7.8) | 739 (7.7) |  |
| Missing | 1634 (9.2) | 51 (0.6) | 1583 (16.5) |  |
| BMI, kg / m^2^ |  |  |  | < 0.001 |
| ＜18.5 | 945 (5.3) | 531 (6.6) | 414 (4.3) |  |
| 18.5-23.9 | 7179 (40.6) | 4356 (53.7) | 2823 (29.4) |  |
| ≥ 24.0 | 5471 (30.9) | 3148 (38.8) | 2323 (24.2) |  |
| Missing | 4110 (23.2) | 72 (0.9) | 4038 (42.1) |  |
| Social isolation |  |  |  | < 0.001 |
| Yes | 9453 (53.4) | 3988 (49.2) | 5465 (56.9) |  |
| No | 8141 (46.0) | 4119 (50.8) | 4022 (41.9) |  |
| Missing | 111 (0.6) | 0 (0.0) | 111 (1.2) |  |
| Body pain |  |  |  | < 0.001 |
| 0 site | 11962 (67.6) | 5412 (66.8) | 6550 (68.2) |  |
| 1-4 sites | 3647 (20.6) | 1716 (21.2) | 1931 (20.1) |  |
| ≥ 5 sites | 1985 (11.2) | 979 (12.1) | 1006 (10.5) |  |
| Missing | 111 (0.6) | 0 (0.0) | 111 (1.2) |  |
| Hypertension |  |  |  | < 0.001 |
| Yes | 4284 (24.2) | 2090 (25.8) | 2194 (22.9) |  |
| No | 13184 (74.5) | 5986 (73.8) | 7198 (75.0) |  |
| Missing | 237 (1.3) | 31 (0.4) | 206 (2.1) |  |
| Diabetes |  |  |  | 0.063 |
| Yes | 993 (5.6) | 487 (6.0) | 506 (5.3) |  |
| No | 16413 (92.7) | 7552 (93.2) | 8861 (92.3) |  |
| Missing | 299 (1.7) | 68 (0.8) | 231 (2.4) |  |
| Heart disease |  |  |  | 0.132 |
| Yes | 2093 (11.8) | 999 (12.3) | 1094 (11.4) |  |
| No | 15372 (86.8) | 7068 (87.2) | 8304 (86.5) |  |
| Missing | 240 (1.4) | 40 (0.5) | 200 (2.1) |  |
| Stroke |  |  |  | < 0.001 |
| Yes | 413 (2.3) | 145 (1.8) | 268 (2.8) |  |
| No | 17111 (96.7) | 7944 (98.0) | 9167 (95.5) |  |
| Missing | 181 (1.0) | 18 (0.2) | 163 (1.7) |  |

*Note*. BMI = body mass index.

**Supplementary Table 5.** Baseline characteristics of participants, stratified by neighborhood environment

| **Characteristics** | **Total** | **Neighborhood environment** | | | ***p* value** |
| --- | --- | --- | --- | --- | --- |
|  |  | **Low-risk (n=2868)** | **Moderate-risk (n=4356)** | **High-risk**  **(n=883)** |  |
| Age, years | 61.4±7.6 | 61.4±7.8 | 61.4±7.5 | 61.2±7.6 | < 0.86 |
| Sex |  |  |  |  | 0.606 |
| Male | 4050 (50.0) | 1412 (49.2) | 2197 (50.4) | 441 (49.9) |  |
| Female | 4057 (50.0) | 1456 (50.8) | 2159 (49.6) | 442 (50.1) |  |
| Residence |  |  |  |  | < 0.001 |
| Rural | 6441 (79.4) | 1807 (63.0) | 3839 (88.1) | 795 (90.0) |  |
| Urban | 1666 (20.6) | 1061 (37.0) | 517 (11.9) | 88 (10.0) |  |
| Marital status |  |  |  |  | 0.685 |
| Married | 6738 (83.1) | 2380 (83.0) | 3632 (83.4) | 726 (82.2) |  |
| Others | 1369 (16.9) | 488 (17.0) | 724 (16.6) | 157 (17.8) |  |
| Education level |  |  |  |  | < 0.001 |
| Low education level | 2349 (29.0) | 611 (21.3) | 1452 (33.3) | 285 (32.3) |  |
| Moderate education level | 3472 (42.8) | 1208 (42.1) | 1847 (42.4) | 417 (47.2) |  |
| High education level | 2286 (28.2) | 1049 (36.6) | 1056 (24.3) | 181 (20.5) |  |
| Smoking status |  |  |  |  | 0.004 |
| Never | 3413 (42.1) | 1718 (59.9) | 2501 (57.4) | 475 (53.8) |  |
| Ever | 4694 (57.9) | 1150 (40.1) | 1855 (42.6) | 408 (46.2) |  |
| Drinking status |  |  |  |  | < 0.001 |
| Never | 2740 (33.8) | 1983 (69.1) | 2796 (62.4) | 588 (66.6) |  |
| Ever | 5367 (66.2) | 885 (30.9) | 1560 (35.8) | 295 (33.4) |  |
| Sleeping status |  |  |  |  | < 0.001 |
| < 6 hours | 2443 (30.1) | 762 (26.6) | 1412 (32.4) | 266 (30.1) |  |
| 6-8 hours | 5020 (61.9) | 1912 (66.7) | 2581 (59.3) | 527 (59.7) |  |
| ＞8 hours | 644 (7.9) | 191 (6.7) | 363 (8.3) | 90 (10.2) |  |
| BMI, kg / m^2^ |  |  |  |  | < 0.001 |
| ＜18.5 | 535 (6.6) | 124 (4.3) | 335 (7.7) | 76 (8.6) |  |
| 18.5-23.9 | 4407 (54.4) | 1409 (49.1) | 2506 (57.6) | 492 (55.7) |  |
| ≥ 24.0 | 3165 (39.0) | 1335 (46.6) | 1515 (34.8) | 315 (35.7) |  |
| Social isolation | 3988 (47.2) | 1273 (44.4) | 2223 (51.0) | 492 (55.7) | < 0.001 |
| Body pain |  |  |  |  | < 0.001 |
| 0 site | 5412 (66.8) | 2123 (74.0) | 2754 (63.2) | 535 (60.6) |  |
| 1-4 sites | 1716 (21.2) | 531 (18.5) | 961 (22.1) | 224 (25.4) |  |
| ≥ 5 sites | 979 (12.1) | 214 (7.5) | 641 (14.7) | 124 (14.0) |  |
| Hypertension | 2096 (25.9) | 796 (27.8) | 1106 (25.4) | 194 (22.0) | 0.002 |
| Diabetes | 449 (5.1) | 198 (6.9) | 248 (5.7) | 42 (4.8) | 0.026 |
| Heart disease | 894 (10.2) | 399 (13.9) | 498 (11.4) | 103 (11.7) | 0.006 |
| Stroke | 145 (1.8) | 45 (1.6) | 81 (1.9) | 19 (2.2) | 0.445 |

*Note*. BMI = body mass index.

**Supplementary Table 6.** Multiplicative and additive interaction of intrinsic capacity and neighborhood environment with dementia risk

| **Interaction term** | **Multiplicative**  **(95% CI)** | **RERI**  **(95% CI)** | **AP**  **(95% CI)** | **SI**  **(95% CI)** |
| --- | --- | --- | --- | --- |
| Low-risk * 0 impairment | Reference | Reference | Reference | Reference |
| Moderate-risk * 1 impairment | 0.80 (0.50, 1.27) | -0.17 (-0.88, 0.5) | -0.08 (-0.43, 0.26) | 0.86 (0.48, 1.53) |
| Moderate-risk * 2 impairments | 1.20 (0.73, 1.95) | 0.20 (-0.61, 1.01) | 0.07 (-0.20, 0.34) | 1.11 (0.71, 1.74) |
| Moderate-risk * 3 impairments | 0.72 (0.44, 1.20) | -0.32 (-1.52, 0.88) | -0.10 (-0.47, 0.28) | 0.88 (0.54, 1.41) |
| Moderate-risk * 4+ impairments | 0.52 (0.30, 0.89) | -1.78 (-3.86, 0.31) | -0.45 (-0.99, 0.09) | 0.62 (0.39, 1.00) |
| High-risk *1 impairment | 1.25 (0.56, 2.7) | 0.78 (-0.55, 2.11) | 0.27 (0.14, 0.68) * | 1.73 (0.60, 5.00) |
| High-risk * 2 impairments | 1.44 (0.66, 3.28) | -1.47 (-3.13, 0.19) | -0.45 (-1.03, 0.13) | 0.61 (0.35, 1.05) |
| High-risk * 3 impairments | 1.88 (0.38, 2.02) | -2.63 (-4.75, -0.51) | -0.76 (-1.52, 0.01) | 0.48, 0.27, 0.87) |
| High-risk * 4+ impairments | 0.61 (0.24, 1.50) | -4.23 (-7.45, -1.01) | -1.04 (-2.18, 0.10) | 0.42 (0.20, 0.87) |

*Note*. CI = confidence interval; RERI = relative excess risk due to interaction; AP = attributable proportion due to interaction; SI = synergy index.

**Supplementary Table 7.** Incremental predictive value of intrinsic capacity and neighborhood environment on dementia risk

|  | **C- statistic**  **(95% CI)** | ***p* value** | **IDI**  **(95% CI)** | ***p* value** | **Continuous NRI**  **(95% CI)** | ***p* value** | **Categorical NRI**  **(95% CI)** | ***p* value** |
| --- | --- | --- | --- | --- | --- | --- | --- | --- |
| Base model | 0.680 (0.658, 0.701) | - | Reference |  | Reference | - | Reference | - |
| Base model + IC | 0.713 (0.691, 0.734) | 0.036 | 0.019 (0.012, 0.029) | < 0.001 | 0.135 (0.089, 0.201) | < 0.001 | 0.127 (0.086, 0.173) | <0.001 |
| Base model + Neighborhood environment | 0.695 (0.673, 0.717) | 0.326 | 0.001 (-0.001, 0.006) | 0.119 | 0.030 (0.001, 0.106) | 0.040 | 0.073 (0.037, 0.120) | < 0.001 |
| Base model + IC + Neighborhood environment | 0.714 (0.692, 0.736) | 0.029 | 0.019 (0.013, 0.029) | < 0.001 | 0.167 (0.107, 0.223) | < 0.001 | 0.134 (0.088, 0.180) | < 0.001 |

*Note*. IC = intrinsic capacity; NRI = net reclassification improvement; IDI = integrated discrimination improvement.

**Supplementary Table 8.** Associations of intrinsic capacity and neighborhood environment with dementia stratified by age, sex, and residence

| **Variables** | **HR (95% CI)** | | | | | |
| --- | --- | --- | --- | --- | --- | --- |
|  | **50-59 years** | **≥ 60 years** | **Male** | **Female** | **Rural** | **Urban** |
| IC impairments |  |  |  |  |  |  |
| 0 | Reference | Reference | Reference | Reference | Reference | Reference |
| 1 | 1.85 (1.32, 2.58) | 1.64 (1.25, 2.15) | 1.82 (1.38, 2.41) | 1.37 (0.96, 1.95) | 1.39 (1.08, 1.80) | 2.45 (1.59, 3.78) |
| 2 | 2.49 (1.75, 3.53) | 2.02 (1.52, 2.68) | 1.88 (1.38, 2.57) | 2.28 (1.61, 3.21) | 1.91 (1.48, 2.47) | 2.76 (1.70, 4.49) |
| 3 | 2.78 (1.87, 4.13) | 2.25 (1.65, 3.05) | 2.31 (1.62, 3.28) | 2.66 (1.85, 3.82) | 2.41 (1.84, 3.17) | 2.45 (1.32, 4.52) |
| 4+ | 3.84 (2.47, 5.97) | 3.09 (2.23, 4.27) | 3.18 (2.16, 4.70) | 3.24 (2.20, 4.78) | 2.79 (2.06, 3.77) | 6.61 (3.62, 12.07) |
| Per 1-point increase | 1.34 (1.23, 1.46) | 1.25 (1.17, 1.33) | 1.26 (1.16, 1.36) | 1.31 (1.22, 1.41) | 1.26 (1.19, 1.34) | 1.41 (1.24, 1.59) |
| Neighborhood environment |  |  |  |  |  |  |
| Low-risk | Reference | Reference | Reference | Reference | Reference | Reference |
| Moderate-risk | 1.55 (1.19, 2.01) | 1.18 (0.98, 1.42) | 1.36 (1.08, 1.70) | 1.17 (0.94, 1.46) | 1.32 (1.10, 1.58) | 1.10 (0.80, 1.51) |
| High-risk | 1.92 (1.35, 2.73) | 1.32 (1.01, 1.73) | 1.47 (1.07, 2.04) | 1.38 (1.01, 1.88) | 1.45 (1.13, 1.86) | 1.35 (0.76, 2.41) |
| Per 1-point increase | 1.13 (1.07, 1.21) | 1.05 (1.01, 1.11) | 1.08 (1.02, 1.15) | 1.07 (1.01, 1.13) | 1.08 (1.03, 1.12) | 1.07 (0.98, 1.17) |

*Note*. IC = intrinsic capacity. All Model was adjusted for age, sex, residence, marital status, education level, drinking status, smoking status, body mass index, sleep time, body pain, hypertension, diabetes, heart disease, and stroke.

**Supplementary Table 9.** Joint associations of intrinsic capacity and neighborhood environment with dementia risk, stratified by age, sex, and residence

| **Neighborhood environment** | **IC impairments** | **HR (95% CI)** | | | | | |
| --- | --- | --- | --- | --- | --- | --- | --- |
|  |  | **50-59 years** | **≥ 60 years** | **Male** | **Female** | **Rural** | **Urban** |
| Low-risk | 0 | Reference | Reference | Reference | Reference | Reference | Reference |
|  | 1 | 1.27 (0.65, 2.49) | 1.99 (1.30, 3.05) | 1.81 (1.15, 2.85) | 1.76 (0.98, 3.18) | 1.33 (0.79, 2.23) | 2.25 (1.36, 3.72) |
|  | 2 | 2.44 (1.26, 4.73) | 1.51 (0.91, 2.50) | 1.10 (0.59, 2.04) | 2.50 (1.38, 4.52) | 1.78 (1.05, 3.01) | 1.72 (0.91, 3.23) |
|  | 3 | 3.40 (1.54, 7.48) | 2.93 (1.79, 4.79) | 2.01 (1.05, 3.83) | 3.89 (2.11, 7.17) | 3.67 (2.19, 6.14) | 1.46 (0.62, 3.43) |
|  | 4+ | 5.25 (2.02, 13.63) | 4.91 (2.74, 8.20) | 4.92 (2.61, 9.27) | 5.13 (2.66, 9.90) | 4.00 (2.23, 7.15) | 8.17 (4.04, 16.49) |
| Moderate-risk | 0 | 1.50 (0.81, 2.79) | 1.37 (0.85, 2.21) | 1.27 (0.78, 2.04) | 1.67 (0.89, 3.13) | 1.58 (0.99, 2.53) | 0.55 (0.21, 1.44) |
|  | 1 | 2.12 (1.18, 3.80) | 1.98 (1.30, 3.03) | 2.14 (1.40, 3.27) | 1.78 (0.99, 3.20) | 2.03 (1.30, 3.16) | 1.67 (0.89, 3.15) |
|  | 2 | 2.92 (1.62, 5.28) | 2.95 (1.93, 4.49) | 2.66 (1.71, 4.15) | 3.37 (1.93, 5.89) | 2.78 (1.78, 4.33) | 3.78 (2.04, 6.99) |
|  | 3 | 4.77 (2.57, 8.84) | 2.63 (1.67, 4.12) | 2.99 (1.84, 4.86) | 3.51 (1.97, 6.26) | 3.13 (1.97, 4.97) | 3.72 (1.24, 5.95) |
|  | 4+ | 4.99 (2.49, 10.02) | 3.45 (2.17, 5.48) | 2.77 (1.60, 4.80) | 4.71 (2.61, 8.51) | 3.68 (2.28, 5.96) | 3.81 (1.63, 8.92) |
| High-risk | 0 | 0.64 (0.15, 2.78) | 1.69 (0.77, 3.68) | 0.98 (0.41, 2.35) | 1.86 (0.62, 5.60) | 1.18 (0.53, 2.62) | 1.28 (0.30, 5.52) |
|  | 1 | 3.79 (1.85, 7.77) | 2.21 (1.20, 4.08) | 2.61 (1.43, 4.75) | 3.00 (1.44, 6.24) | 2.53 (1.45, 4.42) | 4.07 (1.53, 10.80) |
|  | 2 | 3.50 (1.63, 7.49) | 3.09 (1.79, 5.35) | 2.40 (1.28, 4.50) | 4.12 (2.19, 8.03) | 3.20 (1.88, 5.47) | 2.34 (0.78, 7.08) |
|  | 3 | 3.84 (1.60, 9.19) | 3.15 (1.74, 5.72) | 2.45 (1.11, 5.45) | 4.19 (2.10, 8.35) | 3.18 (1.80, 5.65) | 3.45 (0.78, 15.26) |
|  | 4+ | 5.06 (1.63, 15.68) | 3.65 (1.79, 7.43) | 7.45 (3.57, 16.82) | 2.58 (0.98, 6.70) | 3.84 (1.96, 7.54) | 3.71 (0.46, 30.11) |

*Note*. IC = intrinsic capacity. All Model was adjusted for age, sex, residence, marital status, education level, drinking status, smoking status, body mass index, sleep time, body pain, hypertension, diabetes, heart disease, and stroke.

**Supplementary Table 10.** Four-Way decomposition of the effects of neighborhood environment and intrinsic capacity on dementia risk, stratified by age, sex, and residence

| **Comparison Group** | **Effect Component** | **Est (95% CI)** | **SE** | ***p* value** |
| --- | --- | --- | --- | --- |
| **50-59 years** |  |  |  |  |
| Moderate-risk vs low-risk | TE | 0.467 (0.042, 0.892) | 0.217 | 0.031 |
|  | CDE | 0.428 (0.017, 0.839) | 0.210 | 0.041 |
|  | INTref | -0.027 (-0.110, 0.056) | 0.042 | 0.527 |
|  | INTmed | 0.001 (-0.038, 0.040) | 0.020 | 0.957 |
|  | PIE | 0.065 (0.019, 0.111) | 0.024 | 0.006 |
| High-risk vs low-risk | TE | 0.362 (0.079, 0.645) | 0.145 | 0.012 |
|  | CDE | 0.290 (0.012, 0.568) | 0.142 | 0.041 |
|  | INTref | -0.013 (-0.069, 0.042) | 0.028 | 0.639 |
|  | INTmed | 0.005 (-0.027, 0.036) | 0.016 | 0.749 |
|  | PIE | 0.081 (0.035, 0.127) | 0.024 | 0.001 |
| **≥ 60 years** |  |  |  |  |
| Moderate-risk vs low-risk | TE | 0.160 (-0.056, 0.375) | 0.110 | 0.146 |
|  | CDE | 0.138 (0.075, 0.352) | 0.109 | 0.205 |
|  | INTref | -0.023 (-0.054, 0.008) | 0.016 | 0.151 |
|  | INTmed | -0.020 (-0.051, 0.010) | 0.016 | 0.194 |
|  | PIE | 0.064 (0.029, 0.099) | 0.018 | < 0.001 |
| High-risk vs low-risk | TE | 0.173 (0.004, 0.342) | 0.086 | 0.044 |
|  | CDE | 0.161 (0.011, 0.333) | 0.088 | 0.067 |
|  | INTref | -0.020 (-0.050, 0.009) | 0.015 | 0.182 |
|  | INTmed | -0.007 (-0.021, 0.007) | 0.007 | 0.340 |
|  | PIE | 0.039 (0.015, 0.064) | 0.064 | 0.002 |
| **Male** |  |  |  |  |
| Moderate-risk vs low-risk | TE | 0.337 (0.038, 0.451) | 0.153 | 0.027 |
|  | CDE | 0.305 (0.008, 0.429) | 0.157 | 0.044 |
|  | INTref | -0.015 (-0.045, 0.004) | 0.016 | 0.348 |
|  | INTmed | -0.009 (-0.046, 0.009) | 0.019 | 0.655 |
|  | PIE | 0.055 (0.019, 0.087) | 0.019 | 0.003 |
| High-risk vs low-risk | TE | 0.212 (-0.003, 0.427) | 0.110 | 0.053 |
|  | CDE | 0.158 (-0.059, 0.375) | 0.111 | 0.153 |
|  | INTref | 0.014 (-0.020, 0.048) | 0.017 | 0.410 |
|  | INTmed | 0.010 (-0.006, 0.026) | 0.008 | 0.225 |
|  | PIE | 0.030 (0.007, 0.053) | 0.012 | 0.011 |
| **Female** |  |  |  |  |
| Moderate-risk vs low-risk | TE | 0.174 (0.084, 0.433) | 0.132 | 0.018 |
|  | CDE | 0.164 (-0.095, 0.424) | 0.132 | 0.214 |
|  | INTref | -0.036 (-0.091, 0.092) | 0.028 | 0.205 |
|  | INTmed | -0.013 (-0.039, 0.013) | 0.013 | 0.328 |
|  | PIE | 0.059 (0.020, 0.097) | 0.020 | 0.003 |
| High-risk vs low-risk | TE | 0.274 (0.067, 0.480) | 0.105 | 0.009 |
|  | CDE | 0.268 (0.058, 0.478) | 0.107 | 0.012 |
|  | INTref | -0.047 (-0.089, 0.005) | 0.022 | 0.029 |
|  | INTmed | -0.021 (-0.046, 0.003) | 0.013 | 0.090 |
|  | PIE | 0.074 (0.037, 0.111) | 0.019 | < 0.001 |
| **Rural** |  |  |  |  |
| Moderate-risk vs low-risk | TE | 0.358 (0.106, 0.610) | 0.129 | 0.005 |
|  | CDE | 0.353 (0.100, 0.607) | 0.130 | 0.006 |
|  | INTref | -0.042 (-0.084, 0.001) | 0.022 | 0.058 |
|  | INTmed | -0.020 (-0.047, 0.008) | 0.014 | 0.158 |
|  | PIE | 0.066 (0.033, 0.100) | 0.017 | < 0.001 |
| High-risk vs low-risk | TE | 0.247 (0.085, 0.409) | 0.083 | 0.003 |
|  | CDE | 0.219 (0.056, 0.383) | 0.083 | 0.009 |
|  | INTref | -0.022 (-0.053, 0.010) | 0.016 | 0.179 |
|  | INTmed | -0.005 (-0.020, 0.010) | 0.008 | 0.513 |
|  | PIE | 0.054 (0.028, 0.080) | 0.013 | < 0.001 |
| **Urban** |  |  |  |  |
| Moderate-risk vs low-risk | TE | -0.038 (-0.351, 0.277) | 0.161 | 0.813 |
|  | CDE | -0.117 (-0.428, 0.195) | 0.159 | 0.463 |
|  | INTref | 0.014 (-0.054, 0.081) | 0.016 | 0.690 |
|  | INTmed | 0.005 (-0.038, 0.047) | 0.022 | 0.832 |
|  | PIE | 0.060 (0.012, 0.011) | 0.025 | 0.015 |
| High-risk vs low-risk | TE | 0.229 (-0.126, 0.584) | 0.182 | 0.205 |
|  | CDE | 0.195 (-0.171, 0.560) | 0.187 | 0.297 |
|  | INTref | -0.012 (-0.058, 0.036) | 0.024 | 0.631 |
|  | INTmed | -0.007 (-0.057, 0.047) | 0.026 | 0.794 |
|  | PIE | 0.053 (0.008, 0.098) | 0.023 | 0.021 |

*Note*. TE = total effect; CDE = controlled direct effect; INTref = reference interaction; INTmed = mediated interaction; PIE = pure indirect effect. All pathways were adjusted for age, sex, residence, marital status, education level, drinking status, smoking status, body mass index, sleep time, body pain, hypertension, diabetes, heart disease, and stroke.

**Supplementary Table 11**. Sensitivity analysis: Independent associations of intrinsic capacity and neighborhood environment with dementia risk in the complete-case population

| **Variables** | **Event No.** | **HR (95% CI)** | | |
| --- | --- | --- | --- | --- |
|  |  | **Model 1^a^** | **Model 2^b^** | **Model 3^c^** |
| IC impairment index |  |  |  |  |
| 0 | 123/2191 | Reference | Reference | Reference |
| 1 | 239/2429 | 1.71 (1.38, 2.13) | 1.72 (1.38, 2.14) | 1.63 (1.31, 2.03) |
| 2 | 223/1719 | 2.25 (1.80, 2.81) | 2.26 (1.80, 2.83) | 2.06 (1.64, 2.58) |
| 3 | 174/970 | 2.95 (2.33, 3.73) | 2.93 (2.30, 3.74) | 2.50 (1.95, 3.31) |
| 4+ | 130/555 | 3.90 (3.02, 5.03) | 3.95 (3.04, 5.13) | 3.20 (2.45, 4.23) |
| Per 1-point increase | 884/7864 | 1.34 (1.28, 1.41) | 1.34 (1.28, 1.41) | 1.28 (1.24, 1.35) |
| Neighborhood environment |  |  |  | ` |
| Low-risk | 265/2784 | Reference | Reference | Reference |
| Moderate-risk | 505/4217 | 1.27 (1.09, 1.47) | 1.32 (1.13, 1.55) | 1.26 (1.07, 1.47) |
| High-risk | 119/863 | 1.45 (1.17, 1.81) | 1.48 (1.19, 1.86) | 1.44 (1.15, 1.80) |
| Per 1-point increase | 884/7864 | 1.09 (1.05, 1.13) | 1.09 (1.05, 1.14) | 1.08 (1.04, 1.12) |

*Note*: IC = intrinsic capacity. ^a^ Model 1: Adjusted for age and sex. ^b^ Model 2: Adjusted for age, sex, residence, marital status, education level, drinking status, smoking status, BMI, and sleep time. ^c^ Model 3: Additionally adjusted for body pain, hypertension, diabetes, heart disease, and stroke based on Model 2.

**Supplementary Table 12.** Sensitivity analysis: Joint associations of intrinsic capacity and neighborhood environment with dementia risk in the complete-case population

| **Neighborhood environment** | **IC impairments** | **Events No.** | **HR (95% CI)** | | |
| --- | --- | --- | --- | --- | --- |
|  |  |  | **Model 1^a^** | **Model 2^b^** | **Model 3^c^** |
| Low-risk | 0 | 46/951 | Reference | Reference | Reference |
|  | 1 | 85/909 | 1.91 (1.33, 2.73) | 1.91 (1.35, 2.77) | 1.82 (1.27, 2.61) |
|  | 2 | 51/541 | 1.92 (1.29, 2.87) | 1.95 (1.30, 2.91) | 1.78 (1.19, 2.66) |
|  | 3 | 46/243 | 3.39 (2.25, 5.12) | 3.40 (2.57, 5.19) | 2.99 (1.97, 4.56) |
|  | 4+ | 37/140 | 5.29 (3.41, 8.21) | 5.54 (3.55, 8.64) | 4.87 (3.11, 7.64) |
| Moderate-risk | 0 | 67/1060 | 1.31 (0.91, 1.92) | 1.47 (1.01, 2.14) | 1.45 (0.99, 2.12) |
|  | 1 | 123/1266 | 1.96 (1.40, 2.75) | 2.17 (1.54, 3.07) | 2.04 (1.44, 2.88) |
|  | 2 | 136/961 | 2.88 (2.06, 4.23) | 3.22 (2.28, 4.55) | 2.89 (2.04, 4.09) |
|  | 3 | 101/584 | 3.42 (2.41, 4.86) | 3.77 (2.63, 5.41) | 3.15 (2.18, 4.55) |
|  | 4+ | 78/346 | 4.27 (2.95, 6.17) | 4.75 (2.25, 6.95) | 3.76 (2.55, 5.55) |
| High-risk | 0 | 10/180 | 1.19 (0.60, 2.35) | 1.29 (0.65, 2.56) | 1.29 (0.65, 2.56) |
|  | 1 | 31/254 | 2.56 (2.07, 4.96) | 2.85 (1.80, 4.53) | 2.76 (1.74, 4.39) |
|  | 2 | 36/217 | 3.21 (2.10, 5.64) | 3.45 (2.22, 5.38) | 3.23 (2.07, 5.05) |
|  | 3 | 27/143 | 3.62 (2.25, 5.84) | 3.20 (2.41, 6.38) | 3.31 (2.03, 5.42) |
|  | 4+ | 15/69 | 4.48 (2.50, 8.05) | 5.02 (2.77, 9.10) | 3.96 (2.17, 7.23) |

*Note*: IC = intrinsic capacity. ^a^ Model 1: Adjusted for age and sex. ^b^ Model 2: Adjusted for age, sex, residence, marital status, education level, drinking status, smoking status, BMI, and sleep time. ^c^ Model 3: Additionally adjusted for body pain, hypertension, diabetes, heart disease, and stroke based on Model 2.

**Supplementary Table 13.** Four-Way decomposition of the effects of neighborhood environment and intrinsic capacity on dementia risk in the complete-case population

| **Comparison Group** | **Effect Component** | **Est (95% CI)** | **SE** | ***p* value** |
| --- | --- | --- | --- | --- |
| Moderate-risk vs low-risk | TE | 0.258 (0.059, 0.457) | 0.102 | 0.011 |
|  | CDE | 0.236 (0.037, 0.434) | 0.101 | 0.020 |
|  | INTref | -0.025 (-0.056, 0.006) | 0.016 | 0.108 |
|  | INTmed | -0.012 (-0.034, 0.010) | 0.011 | 0.284 |
|  | PIE | 0.059 (0.033, 0.086) | 0.014 | < 0.001 |
| High-risk vs low-risk | TE | 0.261 (0.110, 0.412) | 0.077 | 0.001 |
|  | CDE | 0.230 (0.077, 0.383) | 0.078 | 0.003 |
|  | INTref | -0.016 (-0.040, 0.008) | 0.012 | 0.182 |
|  | INTmed | -0.004 (-0.018, 0.010) | 0.007 | 0.595 |
|  | PIE | 0.051 (0.030, 0.073) | 0.011 | < 0.001 |

*Note*. TE = total effect; CDE = controlled direct effect; INTref = reference interaction; INTmed = mediated interaction; PIE = pure indirect effect. All pathways were adjusted for age, sex, residence, marital status, education level, drinking status, smoking status, body mass index, sleep time, body pain, hypertension, diabetes, heart disease, and stroke.

**Supplementary Table 14.** Sensitivity analysis: independent intrinsic capacity and neighborhood environment with dementia risk, stratified by dementia definition

| **Variables** | **Event No.** | **HR (95% CI)** | | |
| --- | --- | --- | --- | --- |
|  |  | **Model 1^a^** | **Model 2^b^** | **Model 3^c^** |
| **Self-reported cases** |  |  |  |  |
| IC impairment index |  |  |  |  |
| 0 | 95/2243 | Reference | Reference | Reference |
| 1 | 175/2497 | 1.62 (1.27, 2.09) | 1.69 (1.29, 2.13) | 1.57 (1.22, 2.02) |
| 2 | 176/1784 | 2.29 (1.78, 2.95) | 2.36 (1.83, 3.05) | 2.04 (1.65, 2.78) |
| 3 | 117/1005 | 2.57 (1.95, 3.39) | 2.65 (1.99, 3.51) | 2.22 (1.66, 2.97) |
| 4+ | 91/578 | 3.49 (2.59, 4.69) | 3.64 (2.68, 4.94) | 2.89 (2.00, 3.97) |
| Per 1-point increase | 319/8107 | 1.31 (1.23, 1.38) | 1.32 (1.24, 1.40) | 1.25 (1.17, 1.33) |
| Neighborhood environment |  |  |  | ` |
| Low-risk | 204/2868 | Reference | Reference | Reference |
| Moderate-risk | 367/4356 | 1.19 (1.01, 1.41) | 1.31 (1.09, 1.57) | 1.24 (1.04, 1.49) |
| High-risk | 83/883 | 1.33 (1.03, 1.71) | 1.44 (1.11, 1.88) | 1.40 (1.08, 1.83) |
| Per 1-point increase | 654/8107 | 1.07 (1.02, 1.12) | 1.09 (1.04, 1.14) | 1.08 (1.03, 1.13) |
| **Algorithm-identified cases** |  |  |  |  |
| IC impairment index |  |  |  |  |
| 0 | 31/2243 | Reference | Reference | Reference |
| 1 | 87/2497 | 2.39 (1.59, 3.61) | 2.35 (1.56, 3.55) | 2.26 (1.49, 3.42) |
| 2 | 72/1784 | 2.63 (1.72, 4.01) | 2.53 (1.65, 3.88) | 2.36 (1.53, 3.64) |
| 3 | 74/1005 | 4.49 (2.94, 6.88) | 4.20 (2.72, 6.48) | 3.76 (2.41, 5.88) |
| 4+ | 55/578 | 5.14 (3.26, 8.10) | 4.92 (3.09, 7.83) | 4.30 (2.66, 6.94) |
| Per 1-point increase | 319/8107 | 1.38 (1.28, 1.49) | 1.36 (1.26, 1.48) | 1.32 (1.21, 1.44) |
| Neighborhood environment |  |  |  | ` |
| Low-risk | 87/2868 | Reference | Reference | Reference |
| Moderate-risk | 184/4356 | 1.40 (1.09, 1.81) | 1.33 (1.02, 1.73) | 1.28 (0.98, 1.67) |
| High-risk | 48/883 | 1.82 (1.28, 2.59) | 1.65 (1.15, 2.37) | 1.59 (1.11, 2.29) |
| Per 1-point increase | 319/8107 | 1.12 (1.06, 1.20) | 1.10 (1.03, 1.17) | 1.09 (1.02, 1.16) |

*Note*: IC = intrinsic capacity. ^a^ Model 1: Adjusted for age and sex. ^b^ Model 2: Adjusted for age, sex, residence, marital status, education level, drinking status, smoking status, BMI, and sleep time. ^c^ Model 3: Additionally adjusted for body pain, hypertension, diabetes, heart disease, and stroke based on Model 2.

**Supplementary Table 15.** Sensitivity analysis: Joint associations of intrinsic capacity and neighborhood environment with dementia risk, stratified by dementia definition

| **Neighborhood environment** | **IC impairments** | **Events No.** | **HR (95% CI)** | | |
| --- | --- | --- | --- | --- | --- |
|  |  |  | **Model 1^a^** | **Model 2^b^** | **Model 3^c^** |
| **Self-reported case** |  |  |  |  |  |
| Low-risk | 0 | 37/966 | Reference | Reference | Reference |
|  | 1 | 64/946 | 1.75 (1.16, 2.62) | 1.79 (1.19, 2.69) | 1.68 (1.12, 2.52) |
|  | 2 | 44/557 | 2.07 (1.33, 3.20) | 2.15 (1.38, 3.35) | 1.94 (1.25, 3.03) |
|  | 3 | 30/254 | 2.74 (1.69, 4.46) | 2.84 (1.74, 4.64) | 2.46 (1.50, 4.03) |
|  | 4+ | 21/145 | 5.03 (3.07, 8.23) | 5.43 (3.29, 8.96) | 4.71 (2.84, 7.82) |
| Moderate-risk | 0 | 49/1092 | 1.18 (0.77, 1.81) | 1.39 (0.91, 2.15) | 1.37 (0.89, 2.12) |
|  | 1 | 89/1295 | 1.76 (1.20, 2.59) | 2.09 (1.41, 3.09) | 1.95 (1.31, 2.88) |
|  | 2 | 106/1003 | 2.74 (1.89, 3.99) | 3.29 (2.23, 4.84) | 2.92 (1.98, 4.32) |
|  | 3 | 72/604 | 2.99 (2.01, 4.48) | 3.57 (2.36, 5.38) | 2.92 (1.92, 4.44) |
|  | 4+ | 51/362 | 3.39 (2.21, 5.20) | 4.09 (2.60, 6.30) | 3.08 (1.96, 4.85) |
| High-risk | 0 | 9/185 | 1.29 (0.62, 2.66) | 1.50 (0.72, 3.11) | 1.50 (0.72, 3.11) |
|  | 1 | 26/224 | 2.68 (1.34, 3.84) | 2.77 (1.62, 4.73) | 2.69 (1.57, 4.60) |
|  | 2 | 36/217 | 2.88 (1.74, 4.75) | 3.34 (2.01, 5.57) | 3.12 (1.87, 5.21) |
|  | 3 | 15/147 | 2.53 (1.39, 4.62) | 2.99 (1.62, 5.53) | 2.47 (1.33, 4.58) |
|  | 4+ | 11/71 | 4.12 (2.10, 8.10) | 4.92 (2.47, 9.80) | 3.80 (1.90, 7.61) |
| **Algorithm-identified cases** | |  |  |  |  |
| Low-risk | 0 | 12/966 | Reference | Reference | Reference |
|  | 1 | 28/946 | 2.26 (1.45, 4.44) | 2.25 (1.14, 4.42) | 2.16 (1.09, 4.25) |
|  | 2 | 13/557 | 1.68 (0.76, 3.68) | 1.63 (0.74, 3.58) | 1.54 (0.70, 3.40) |
|  | 3 | 21/254 | 5.60 (2.74, 11.42) | 5.22 (2.54, 10.71) | 4.74 (2.29, 9.81) |
|  | 4+ | 13/145 | 5.15 (2.33, 11.42) | 5.02 (2.25, 11.20) | 4.54 2.02, 10.20) |
| Moderate-risk | 0 | 18/1092 | 1.35 (0.65, 2.81) | 1.35 (0.65, 2.82) | 1.34 (0.64, 2.78) |
|  | 1 | 46/1295 | 2.77 (1.47, 5.22) | 2.71 (1.42, 5.17) | 2.60 (1.36, 4.97) |
|  | 2 | 46/1003 | 2.39 (1.79, 6.40) | 3.32 (1.74, 6.36) | 3.07 (1.59, 5.90) |
|  | 3 | 39/604 | 4.44 (2.32, 8.51) | 4.26 (2.19, 8.29) | 3.81 (1.94, 7.48) |
|  | 4+ | 35/362 | 5.94 (3.06, 11.54) | 5.81 (2.94, 11.47) | 5.03 (2.52, 10.06) |
| High-risk | 0 | 1/185 | 0.46 (0.06, 3.23) | 0.43 (0.06, 3.34) | 0.44 (0.06, 3.35) |
|  | 1 | 13/224 | 4.00 (1.82, 8.76) | 3.86 (1.75, 8.55) | 3.71 (1.68, 8.23) |
|  | 2 | 15/147 | 4.28 (1.95, 9.40) | 4.22 (1.82, 8.91) | 3.78 (1.70, 8.40) |
|  | 3 | 14/147 | 6.59 (3.04, 14.39) | 6.15 (2.79, 13.54) | 5.50 (2.48, 12.20) |
|  | 4+ | 7/71 | 6.19 (4.23, 15.78) | 5.93 (2.29, 15.35) | 5.14 (1.97, 13.42) |

*Note*: IC = intrinsic capacity. ^a^ Model 1: Adjusted for age and sex. ^b^ Model 2: Adjusted for age, sex, residence, marital status, education level, drinking status, smoking status, BMI, and sleep time. ^c^ Model 3: Additionally adjusted for body pain, hypertension, diabetes, heart disease, and stroke based on Model 2.

**Supplementary Table 16.** Four-Way decomposition of the effects of neighborhood environment and intrinsic capacity on dementia risk​, stratified by dementia definition

| **Comparison Group** | **Effect Component** | **Est (95% CI)** | **SE** | ***p* value** |
| --- | --- | --- | --- | --- |
| **Self-reported cases** |  |  |  |  |
| Moderate-risk vs low-risk | TE | 0.254 (0.026, 0.483) | 0.117 | 0.029 |
|  | CDE | 0.237 (0.009, 0.465) | 0.117 | 0.042 |
|  | INTref | -0.026 (-0.058, 0.008) | 0.017 | 0.130 |
|  | INTmed | -0.014 (-0.040, 0.010) | 0.013 | 0.267 |
|  | PIE | 0.057 (0.030, 0.085) | 0.014 | < 0.001 |
| High-risk vs low-risk | TE | 0.238 (0.066, 0.411) | 0.088 | 0.007 |
|  | CDE | 0.220 (0.044, 0.396) | 0.090 | 0.014 |
|  | INTref | -0.021 (-0.048, 0.005) | 0.014 | 0.117 |
|  | INTmed | -0.008 (-0.024, 0.008) | 0.008 | 0.306 |
|  | PIE | 0.048 (0.025, 0.070) | 0.012 | < 0.001 |
| **Algorithm-identified cases** |  |  |  |  |
| Moderate-risk vs low-risk | TE | 0.255 (0.088, 0.598) | 0.175 | 0.046 |
|  | CDE | 0.221 (-0.124, 0.566) | 0.176 | 0.209 |
|  | INTref | -0.021 (-0.076, 0.034) | 0.028 | 0.456 |
|  | INTmed | -0.007 (-0.043, 0.029) | 0.018 | 0.694 |
|  | PIE | 0.062 (0.026, 0.098) | 0.018 | 0.001 |
| High-risk vs low-risk | TE | 0.296 (0.036, 0.557) | 0.133 | 0.026 |
|  | CDE | 0.241 (0.025, 0.507) | 0.136 | 0.076 |
|  | INTref | -0.001 (-0.040, 0.040) | 0.020 | 0.993 |
|  | INTmed | -0.007 (-0.014, 0.027) | 0.011 | 0.533 |
|  | PIE | 0.049 (0.019, 0.078) | 0.015 | 0.001 |

*Note*. TE = total effect; CDE = controlled direct effect; INTref = reference interaction; INTmed = mediated interaction; PIE = pure indirect effect. All pathways were adjusted for age, sex, residence, marital status, education level, drinking status, smoking status, body mass index, sleep time, body pain, hypertension, diabetes, heart disease, and stroke.

**Supplementary Table 17.** Sensitivity analysis: independent associations of neighborhood environment and intrinsic capacity with dementia risk, after excluding participants with new-onset dementia during wave 2 and wave 3

| **Variables** | **Event No.** | **HR (95% CI)** | | |
| --- | --- | --- | --- | --- |
|  |  | **Model 1^a^** | **Model 2^b^** | **Model 3^c^** |
| IC impairment index |  |  |  |  |
| 0 | 103/2222 | Reference | Reference | Reference |
| 1 | 189/2445 | 1.61 (1.27, 2.05) | 1.61 (1.27, 2.05) | 1.54 (1.21, 1.97) |
| 2 | 194/1747 | 2.31 (1.82, 2.94) | 2.33 (1.83, 2.98) | 2.15 (1.67, 2.75) |
| 3 | 142/969 | 2.83 (2.18, 3.66) | 2.84 (2.18, 3.70) | 2.45 (1.86, 3.22) |
| 4+ | 91/534 | 3.33 (2.49, 4.45) | 3.39 (2.52, 4.57) | 2.81 (2.06, 3.82) |
| Per 1-point increase | 719/7917 | 1.32 (1.25, 1.39) | 1.32 (1.25, 1.40) | 1.26 (1.19, 1.34) |
| Neighborhood environment |  |  |  |  |
| Low-risk | 202/2798 | Reference | Reference | Reference |
| Moderate-risk | 419/4257 | 1.36 (1.15, 1.61) | 1.44 (1.20, 1.71) | 1.38 (1.15, 1.64) |
| High-risk | 98/862 | 1.57 (1.23, 1.99) | 1.61 (1.26, 2.07) | 1.57 (1.26, 2.02) |
| Per 1-point increase | 719/7917 | 1.10 (1.05, 1.15) | 1.11 (1.06, 1.16) | 1.10 (1.05, 1.14) |

*Note*: IC = intrinsic capacity. ^a^ Model 1: Adjusted for age and sex. ^b^ Model 2: Adjusted for age, sex, residence, marital status, education level, drinking status, smoking status, BMI, and sleep time. ^c^ Model 3: Additionally adjusted for body pain, hypertension, diabetes, heart disease, and stroke based on Model 2.

**Supplementary Table 18.** Sensitivity analysis: Joint associations of intrinsic capacity and neighborhood environment with dementia risk, after excluding participants with new-onset dementia during wave 2 and wave 3

| **Neighborhood environment** | **IC impairments** | **Events No.** | **HR (95% CI)** | | |
| --- | --- | --- | --- | --- | --- |
|  |  |  | **Model 1^a^** | **Model 2^b^** | **Model 3^c^** |
| Low-risk | 0 | 37/956 | Reference | Reference | Reference |
|  | 1 | 61/921 | 1.67 (1.11, 2.51) | 1.68 (1.12, 2.53) | 1.60 (1.06, 2.41) |
|  | 2 | 46/551 | 2.15 (1.39, 3.31) | 2.18 (1.41, 3.37) | 2.00 (1.29, 3.11) |
|  | 3 | 38/244 | 3.40 (2.15, 5.36) | 3.43 (2.16, 5.43) | 3.04 (1.91, 4.85) |
|  | 4+ | 20/126 | 3.70 (2.14, 6.41) | 3.98 (2.28, 6.93) | 3.61 (2.07, 6.33) |
| Moderate-risk | 0 | 58/1083 | 1.40 (0.93, 2.11) | 1.55 (1.02, 2.35) | 1.54 (1.01, 2.23) |
|  | 1 | 101/1272 | 1.98 (1.36, 2.88) | 2.19 (1.49, 3.22) | 2.08 (1.41, 3.05) |
|  | 2 | 118/978 | 3.02 (2.09, 4.38) | 3.40 (2.33, 4.97) | 3.10 (2.11, 4.55) |
|  | 3 | 79/580 | 3.23 (2.18, 4.79) | 3.63 (2.42, 5.44) | 3.09 (2.05, 4.67) |
|  | 4+ | 63/344 | 4.27 (2.84, 6.45) | 4.79 (2.14, 7.32) | 3.86 (2.50, 5.95) |
| High-risk | 0 | 8/138 | 1.16 (0.54, 2.49) | 1.25 (0.58, 2.70) | 1.26 (0.59, 2.72) |
|  | 1 | 27/252 | 2.78 (1.69, 4.56) | 3.10 (1.83, 5.13) | 3.04 (1.83, 5.03) |
|  | 2 | 30/218 | 3.26 (2.01, 5.28) | 3.54 (2.17, 5.78) | 3.39 (2.07, 5.05) |
|  | 3 | 25/145 | 4.08 (2.45, 6.80) | 4.51 (2.67, 7.60) | 3.86 (2.28, 6.55) |
|  | 4+ | 8/61 | 3.07 (1.42, 6.60) | 3.41 (1.57, 7.42) | 2.74 (1.25, 5.97) |

*Note*: IC = intrinsic capacity. ^a^ Model 1: Adjusted for age and sex. ^b^ Model 2: Adjusted for age, sex, residence, marital status, education level, drinking status, smoking status, BMI, and sleep time. ^c^ Model 3: Additionally adjusted for body pain, hypertension, diabetes, heart disease, and stroke based on Model 2.

**Supplementary Table 19.** Four-Way decomposition of the effects of neighborhood environment and intrinsic capacity on dementia risk​, after excluding participants with new-onset dementia during wave 2 and wave 3

| **Comparison Group** | **Effect Component** | **Est (95% CI)** | **SE** | ***p* value** |
| --- | --- | --- | --- | --- |
| Moderate-risk vs low-risk | TE | 0.368 (0.122, 0.607) | 0.124 | 0.003 |
|  | CDE | 0.333 (0.091, 0.574) | 0.123 | 0.007 |
|  | INTref | -0.019 (-0.051, 0.013) | 0.016 | 0.233 |
|  | INTmed | -0.012 (-0.033, 0.021) | 0.014 | 0.648 |
|  | PIE | 0.059 (0.030, 0.087) | 0.014 | < 0.001 |
| High-risk vs low-risk | TE | 0.303 (0.130, 0.477) | 0.086 | 0.001 |
|  | CDE | 0.278 (0.102, 0.455) | 0.091 | 0.002 |
|  | INTref | -0.015 (-0.038, 0.008) | 0.012 | 0.203 |
|  | INTmed | -0.004 (-0.020, 0.011) | 0.008 | 0.593 |
|  | PIE | 0.044 (0.022, 0.066) | 0.011 | < 0.001 |

*Note*. TE = total effect; CDE = controlled direct effect; INTref = reference interaction; INTmed = mediated interaction; PIE = pure indirect effect. All pathways were adjusted for age, sex, residence, marital status, education level, drinking status, smoking status, body mass index, sleep time, body pain, hypertension, diabetes, heart disease, and stroke.

**Supplementary Table 20.** E-values for the association of intrinsic capacity and neighborhood environment with dementia risk

| **Variables** | **Event No.** | **E-value (95% CI)** | | |
| --- | --- | --- | --- | --- |
|  |  | **Model 1^a^** | **Model 2^b^** | **Model 3^c^** |
| IC impairments |  |  |  |  |
| 0 | 123/2191 | Reference | Reference | Reference |
| 1 | 239/2429 | 2.81 (2.10, NA) | 2.83 (2.10, NA) | 2.64 (1.95, NA) |
| 2 | 223/1719 | 3.93 (3.00, NA) | 3.95 (3.00, NA) | 3.54 (2.66, NA) |
| 3 | 174/970 | 5.35 (4.09, NA) | 5.31 (4.03, NA) | 4.44 (3.31, NA) |
| 4+ | 130/555 | 7.26 (5.49, NA) | 7.36 (5.53, NA) | 5.85 (4.33, NA) |
| Per 1-point increase | 884/7864 | 2.01 (1.88, NA) | 2.01 (1.88, NA) | 1.88 (1.79, NA) |
| Neighborhood environment |  |  |  |  |
| Low-risk | 265/2784 | Reference | Reference | Reference |
| Moderate-risk | 505/4217 | 1.86 (1.40, NA) | 1.97 (1.51, NA) | 1.83 (1.34, NA) |
| High-risk | 119/863 | 2.26 (1.62, NA) | 2.32 (1.67, NA) | 2.24 (1.57, NA) |
| Per 1-point increase | 884/7864 | 1.40 (1.28, NA) | 1.40 (1.28, NA) | 1.38 (1.24, NA) |

*Note*. IC = intrinsic capacity.

**Supplementary Table 21.** Independent association of intrinsic capacity with dementia risk after merging sensory domains (version and hearing items)

| **IC impairment index** | **Event No.** | **HR (95% CI)** | | |
| --- | --- | --- | --- | --- |
|  |  | **Model 1^a^** | **Model 2^b^** | **Model 3^c^** |
| 0 | 192/3093 | Reference | Reference | Reference |
| 1 | 323/2800 | 1.82 (1.52, 2.17) | 1.84 (1.53, 2.20) | 1.74 (1.45, 2.09) |
| 2 | 228/1470 | 2.33 (1.92, 2.84) | 2.34 (1.91, 2.86) | 2.08 (1.69, 2.55) |
| 3 | 125/580 | 3.31 (2.63, 4.17) | 3.35 (2.64, 4.25) | 2.81 (2.20, 3.59) |
| 4+ | 41/164 | 3.67 (2.60, 5.18) | 3.69 (2.60, 5.24) | 3.04 (2.12, 4.35) |
| Per 1-point increase | 909/8107 | 1.42 (1.34, 1.51) | 1.42 (1.34, 1.51) | 1.34 (1.26, 1.43) |

^a^ Model 1: Adjusted for age and sex. ^b^ Model 2: Adjusted for age, sex, residence, marital status, education level, drinking status, smoking status, BMI, and sleep time. ^c^ Model 3: Additionally adjusted for body pain, hypertension, diabetes, heart disease, and stroke based on Model 2.

**Supplementary Table 22.** Independent association of weighted-score-based neighborhood environment with dementia risk

| **Neighborhood environment** | | **Event No.** | **HR (95% CI)** | | |
| --- | --- | --- | --- | --- | --- |
|  |  |  | **Model 1^a^** | **Model 2^b^** | **Model 3^c^** |
| Low-risk | 251/2746 | | Reference | Reference | Reference |
| Moderate-risk | 320/2677 | | 1.29 (1.10, 1.52) | 1.35 (1.14, 1.60) | 1.29 (1.09, 1.53) |
| High-risk | 338/2684 | | 1.38 (1.17, 1.62) | 1.44 (1.21, 1.71) | 1.36 (1.14, 1.62) |

^a^ Model 1: Adjusted for age and sex. ^b^ Model 2: Adjusted for age, sex, residence, marital status, education level, drinking status, smoking status, BMI, and sleep time. ^c^ Model 3: Additionally adjusted for body pain, hypertension, diabetes, heart disease, and stroke based on Model 2.


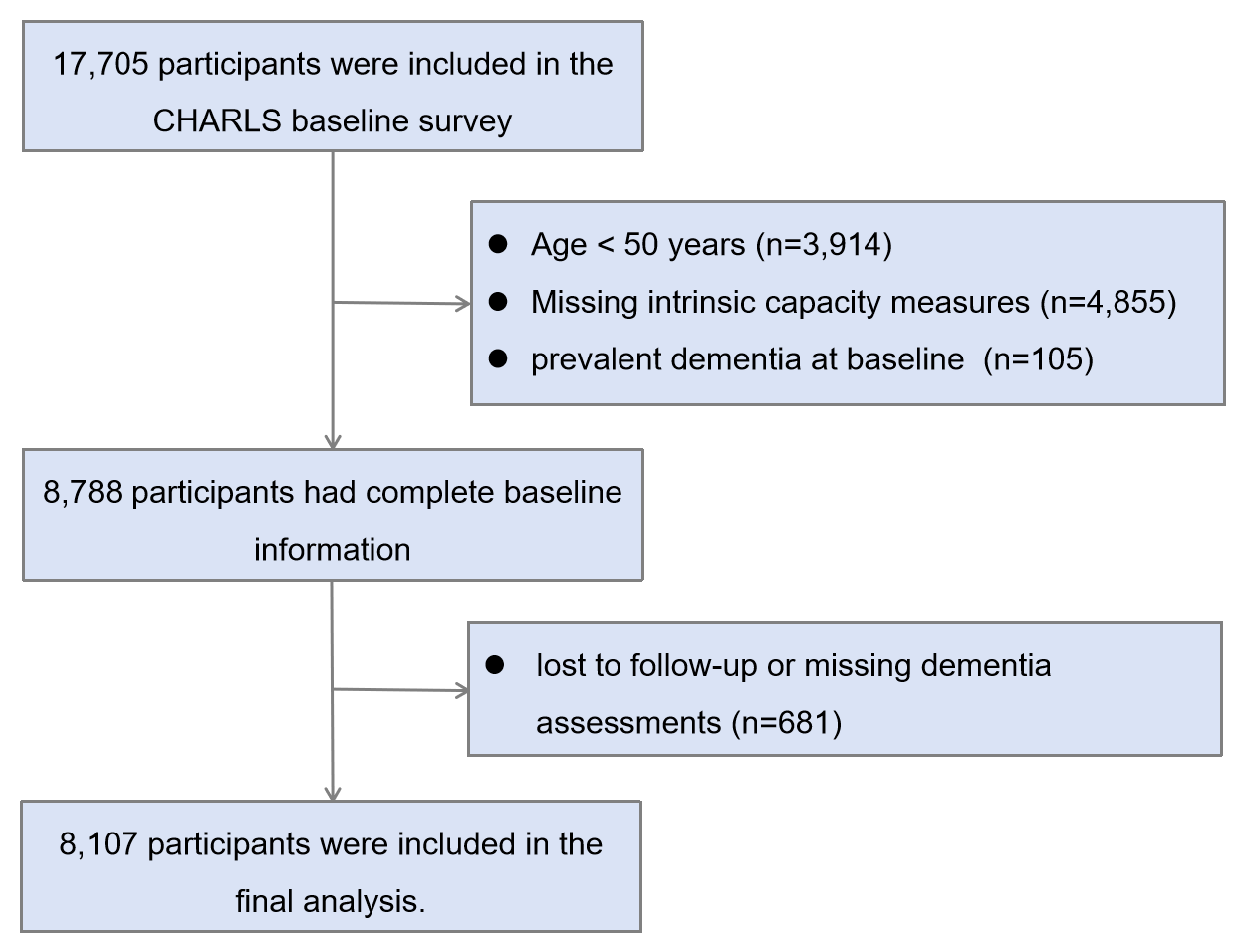


**Supplementary Figure 1.** Selection process of the study participants


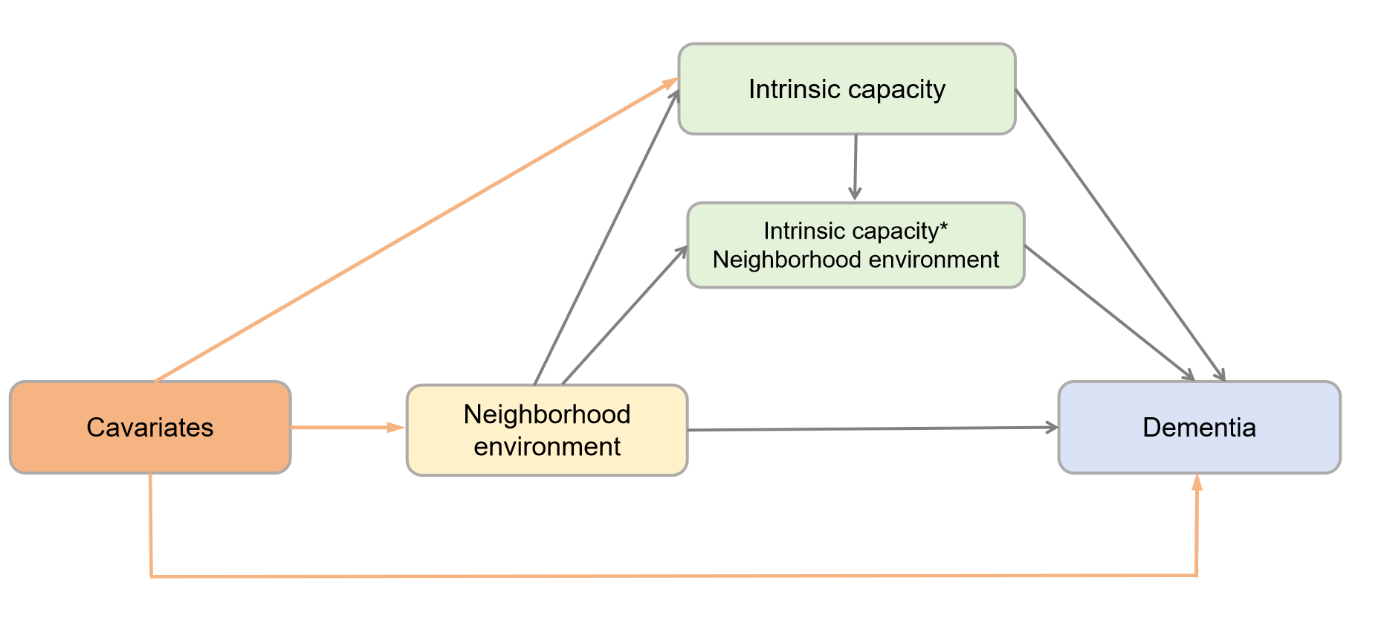


**Supplementary Figure 2.** Directed acyclic graph for the association of neighborhood environment and intrinsic capacity with dementia risk.​
